# Refined analysis of microplastics in endemic fish species from Lake Victoria using µ-FTIR and pyrolysis GC-MS

**DOI:** 10.1007/s10661-026-15765-5

**Published:** 2026-08-14

**Authors:** Timothy Omara, Barbora Benetková, Ivan Sumerskii, Patrick Ssebugere, Christine Kyarimpa, Solomon Omwoma Lugasi, Thomas Rosenau, Christine Betty Nagawa, Stefan Böhmdorfer

**Affiliations:** 1 Institute of Chemistry of Renewable Resources, Department of Natural Sciences and Sustainable Resources, BOKU University, Konrad-Lorenz-Straße 24, 3430 Tulln, Austria; 2 Department of Chemistry, College of Natural Sciences, Makerere University, P.O. Box 7062, Kampala, Uganda; 3 Core Facility Analysis of Lignocellulosics (ALICE), BOKU University, Konrad-Lorenz-Straße 24, 3430 Tulln, Austria; 4 Department of Chemistry, Faculty of Science, Kyambogo University, P.O. Box 1, Kampala, Uganda; 5 Department of Physical Sciences, Jaramogi Oginga Odinga University of Science and Technology, P.O. Box 210-40601, Bondo, Kenya; 6 Johan Gadolin Process Chemistry Centre, Åbo Akademi University, Porthansgatan 3, 20500 Åbo/Turku, Finland; 7 Department of Forestry, Biodiversity and Nature Conservation, College of Agricultural and Environmental Sciences, Makerere University, P.O. Box 7062, Kampala, Uganda

**Keywords:** Backflush, Microplastics, Nylon, *Protopterus aethiopicus*, *Synodontis victoriae*, Lake Victoria

## Abstract

Pyrolysis–gas chromatography–mass spectrometry (Pyr-GC-MS) has evolved into one of the most powerful methods for microplastics (MPs) analysis. However, analytical challenges are still encountered whenever Pyr-GC-MS is applied to matrices with high organic matter, protein and lipid contents. In this study, a workflow integrating stereomicroscopy, micro-Fourier transform infrared (µ-FTIR) spectroscopy and Pyr-GC-MS was extended to analyse MPs in three endemic fish species (*Protopterus aethiopicus*, *Rastrineobola argentea* and *Synodontis victoriae*) from Lake Victoria. Stereomicroscopic analysis only detected MPs in the gastrointestinal tracts of *P. aethiopicus* and *S. victoriae*, with the highest mean particle numbers being 16.0 ± 7.8 and 14.7 ± 6.7 items/fish taxon, respectively. No MPs were found in whole samples of *R. argentea*. The microparticles were mostly blue and brown 0.3–1.9-mm fragments and filaments, which µ-FTIR analysis confirmed to be composed of nylon 66, nylon 6/66, nylon 6/10, nylon 11, polyethylene and polypropylene. Retention time locking and post-column backflush considerably reduced the cycle time and increased stability of the Pyr-GC-MS method. Nylon 66 (243 µg/g in *S. victoriae*), nylon 6 (0.24–96 µg/g), polypropylene (6.4 µg/g in *P. aethiopicus*), poly(ethylene terephthalate) (1.2–107 µg/g) and styrene-butadiene rubber (8.5–34.8 µg/g) were quantified. Polyethylene and poly(methyl methacrylate) were detected below their limits of quantification. To prevent false positive detection of polyethylene in the lipid-rich tissues, the evaluation focussed on C21-α,ω-alkene as a marker. Polypropylene was not detected in some samples, as it formed several discrete propylene oligomers during pyrolysis, which suppressed the yield of its target quantification marker (2,4-dimethyl-1-heptene).

**Supplementary Information:**

The online version contains supplementary material available at https://doi.org/10.1007/s10661-026-15765-5.

## Introduction

The size-defined term “microplastics” (MPs) was first coined in 2004 to describe small plastic debris of granular and fibrous nature (Thompson et al., 2004). The current definition of MPs has encompassed all water-insoluble synthetic plastic debris or polymeric matrices measuring 1–5000 µm in size. The particles are also likely to contain additives (International Organization for Standardization, 2023). The initial focus of research on MPs was placed on the marine environment. It took a few years to appreciate the role of freshwater lakes and rivers as reservoirs and transportation routes of MPs between terrestrial sources and the marine environment (Mutshekwa et al., 2025).

Lake Victoria, the largest of the seven African Great Lakes, is a freshwater resource that drains into the Mediterranean Sea through the Nile River. Its ecosystem and the surrounding satellite lakes in East Africa are a unique biodiversity hotspot with fish species that are members of the Trans-Saharan assemblage (Mwanja et al., 2001). The lake once supported an exceptionally rich and diverse community of cichlid fishes, with over 200 endemic species in the sublittoral and offshore areas (Okechi et al., 2022). However, human activities such as discharge of untreated industrial and agricultural waste, rapid urbanisation and plastic waste pollution continue to threaten this biodiversity, making Lake Victoria a classic example of a conservation paradox (Bwire & Alhassan, 2025; Masese et al., 2025).

To date, some endemic and introduced fish species (*Clarias gariepinus*, *Oreochromis niloticus*, *Synodontis victoriae* and *Lates niloticus*) in Lake Victoria have been reported to ingest MPs (Atukwatse et al., 2026; Gathu et al., 2024; Nicholson, 2021). However, the spatial and temporal variations, potential biomagnification and trophic transfer of MPs among fish species of Lake Victoria (L. Victoria) remain largely unknown. The native shoaling minnow (*Rastrineobola argentea*), for example, has never been investigated for its potential contamination with MPs, even though it is a keystone species and a popular delicacy among the riparian communities of the L. Victoria basin. Since such small fish are consumed whole and in large quantities (Wessels et al., 2023), transfer of MPs from them to humans can be higher than from larger fish where only muscle tissues are consumed. *Rastrineobola argentea* (*R. argentea*) is also known to exhibit diel vertical migratory behaviour (Goudswaard et al., 2004), which could facilitate the redistribution of MPs to deeper layers of the lake and sediments.

Earlier studies of MPs in L. Victoria used particle-based techniques (stereomicroscopy and micro-Fourier transform infrared spectroscopy) which are useful for initial MPs characterisation, but are barely suitable for microparticles ≤ 10 µm in size (Ciornii et al., 2025). In contrast, particle-size independent thermoanalytical techniques (pyrolysis, thermal extraction and desorption coupled with gas chromatography–mass spectrometry) have additional benefits for MPs analysis, as they allow simultaneous identification and quantification of multiple polymers with minimum to no sample preparation (Rauert et al., 2022; Sefiloglu et al., 2024). Still, in complex matrices such as sewage sludge and biota, microplastic isolation and analysis is challenging because of the high content of lipids and proteins which can obscure chromatographic and spectral features of microplastic polymers or alter their pyrolysate signals (Almeida et al., 2026; Jeffries et al., 2025; Witzig et al., 2020). Thus, efficient isolation and complementary analytical techniques are necessary for reliable MPs characterisation, and to reduce the risk of non-detection and false positives (Rauert et al., 2022; Ribeiro et al., 2020).

In a previous study, we investigated the suitability of micro-Fourier transform infrared (µ-FTIR) spectroscopy and pyrolysis–gas chromatography–mass spectrometry (Pyr-GC-MS) for complementary analysis of MPs in eutrophic surface water trawled from L. Victoria (Omara et al., 2026). In continuity, the present study focussed on MPs in three endemic fish species (*Protopterus aethiopicus*, *R. argentea* and *Synodontis victoriae*) sampled from L. Victoria. The challenging matrix of animal tissues demanded adjustments to the analytical workflow. µ-FTIR was expanded by secondary confirmation of polymer composition using KnowItAll software. The Pyr-GC-MS method was developed further by locking the retention time to styrene and incorporating a post-run backflush for a reliable confirmation of the contained polymers and a higher robustness of the analytical setup. This adjustment enabled the calibration previously established for eutrophic surface water samples from L. Victoria to be transferred to the fish samples, reducing the analysis time, cost and resource burden associated with repetitive univariate calibration.

## Materials and methods

### Standards, reference materials and chemicals

The MPs calibration set (MPs-SiO_2_ and MPs-CaCO_3_ standards, SiO_2_ and CaCO_3_ diluents) and polystyrene reference material (2.5 mg as thin film with 5 wt % methyl stearate) were supplied by Frontier Laboratories Ltd (Fukushima, Japan). Bulk solvents and reagents: acetone (≥ 99.8%, Honeywell, Saint-Germain-en-Laye, France), toluene (≥ 99.7%, Sigma-Aldrich, Rehovot, Israel), zinc chloride (> 98%, Sigma-Aldrich, Schnelldorf, Germany) and potassium hydroxide (85%, Loba Chemie Pvt Ltd, Mumbai, India) were used as supplied, without any further purification.

### Sampling

Sampling was done during the dry and wet seasons (August and November 2023) from Ripon Falls (0° 24′ 52″ N 33° 12′ 18″ E), Katosi (0° 9′ 5″ N 32° 48′ 6″ E) and Port Bell (0°17′23.5″N 32°39′18.2″E) fish landing beaches (FLBs) of L. Victoria. Study site selection was guided by geographical settings as well as the fish landing systems. Katosi is the busiest, rural and the most commercially organised site, followed by Port Bell (an industrial area and a freight port) and Ripon Falls, which has many recreational beaches, various tourist activities and a nearby slum settlement (Omara et al., 2026).

The endemic fish species sampled (*Protopterus aethiopicus*, *R. argentea* and *Synodontis victoriae*) are of commercial relevance. Of those, *R. argentea* is the most important fishery by mass after Nile perch (*Lates niloticus*), with an estimated volume of 166 thousand tons consumed in 2014 (Clarke et al., 2022). With the exception of *R. argentea* which is agastric, planktivorous and a dominant component of L. Victoria’s food web, the other gastric species belong to the same feeding guild (Supplementary Table S1). A total of 54 samples of different fish species (*n* = 3 samples per FLB, taxon and season) were obtained from fishermen using baited nets, hooks and 5-mm mesh-sized lampara nets (Clarke et al., 2022). The wet weights, total and standard lengths of the blot-dried samples were measured.

### Microplastic extraction and isolation

With the exception of *R. argentea*, all the samples were dissected using a pair of stainless-steel scissors and then filleted. The gastrointestinal tracts (GIT) of *P. aethiopicus* and *S. victoriae*, and gills of *S. victoriae* were removed, weighed and then transferred into 500 mL glass beakers. To the GIT, gills, edible muscles (10 g) and whole *R. argentea*, 10% of potassium hydroxide solution was added (5:1 v/w) and incubated in an oven at 60 °C for 19–24 h (Pradit et al., 2023). Digested samples were filtered through stacked 5.0 and 0.25-mm stainless-steel sieves and the retentates of the 0.25-mm sieve were backwashed with distilled water into 1 L glass beakers. To each resultant solution, 50 mL of saturated zinc chloride solution (1.7 g/mL) were added, and the mixture was stirred for 15 min before it was transferred into a density separator. The samples were left to stand for 1 h at room temperature. Floating MPs were filtered through glass microfibre filters (GF/C, Ø 47 mm). The filters were transferred into glass petri dishes, covered loosely with aluminium foil and left to dry in air.

### Physical characteristics of extracted MPs

The filters were screened under a stereomicroscope (ZEISS Stemi 508; Carl Zeiss microscopy GmbH, Jena, Germany) at up to 50-fold magnification. The microscope was equipped with an Axiocam 208 colour camera controlled by Labscope imaging software. Suspected particles were subjected to break test and recorded either as items/fish or standardised to items/g of whole fish, GIT, gill or edible muscle. Particle colours, morphologies and size categories (0.3–0.9 mm, 1.0–1.9 mm, 2.0–2.9 mm, 3.0–3.9 mm and 4.0–4.9 mm) were also recorded (Egessa et al., 2020). For morphologies, fragments were identified as irregular, non-linear particles with a low length-to-width ratio (< 3). Fibres were thin, flexible, thread-like particles with a uniform width and high length-to-width ratio (≥ 3). Filaments were elongated, thread- or cord-like particles with a high length-to-width ratio (≥ 3) that appeared thicker and more rigid than fibres (Ateia et al., 2022; Bošković et al., 2022).

### Micro-Fourier transform infrared spectroscopy of microparticles

A subset of filters with MPs (*n* = 12) were randomly selected and subjected to µ-FTIR spectroscopy using an IRTracer-100/AIM-9000 infrared microscope (Shimadzu Corporation, Kyoto, Japan) (Ocakacon et al., 2025). Initial polymer identification was achieved through matching the unprocessed spectrum against reference spectra in the polymer library of LabSolutions IR. MPs were assigned to the highest scoring polymer hit that was a synthetic plastic. Hit quality index (HQI) scores > 70% were considered successful hits. Spectra of particles with 60–70% similarity to known peaks of standard polymers were first overlaid and examined in Peak Spectroscopy software (version 4.00, Build 524; Operant LLC, Monona, WI, USA) within a wavenumber tolerance of ± 10 cm^−1^. They were then exported as CSV files into KnowItAll software (Analytical Edition, version 25.1.67.0, Wiley Science Solutions, Hoboken, NJ, USA) and matched against ATR-IR Sadtler Polymers and Monomers libraries by functional group. Matches scoring < 60% in all the libraries were excluded from further processing.

### Quantitative Pyr-GC-MS analysis of MPs

The 12 filters subjected to µ-FTIR spectroscopy were excluded from Pyr-GC-MS analysis since µ-FTIR required prolonged focusing and handling, which increases the risk of contamination. Their corresponding duplicate filters were instead analysed by Pyr-GC-MS (details of the filters allocated to µ-FTIR and Pyr-GC-MS are provided in Supplementary Table S2). Thus, eight circular discs were punched from each of the remaining 24 filters on a cutting mat using 4-mm micropunchers (Frontier Laboratories Ltd, Japan) following a quarter-judgmental hybrid sampling method. A pair of discs were fitted into 80 µL pyrolysis cups and transferred to the pyrolyser auto-shot sampler. The analytical conditions were based on earlier work (Omara et al., 2026) and are summarised in Table 1.

**Table 1:** Analytical conditions used for quantitative analysis of microplastics by pyrolysis GC-MS

Analytical unit	Instrumentation/analytical conditions
**1. Pyrolysis unit**	
Instrument	Micro-furnace pyrolyser (EGA/PY-3030D) ^*a*^
Sample injection	Auto-shot sampler (AS-1020E) ^*a*^
Analysis mode	Online, single shot (flash pyrolysis)
Pyrolysis temperature (time)	600 °C (1 min)
Carrier gas	Helium
Interface temperature	300 °C
**2. Gas chromatograph**	
Instrument	Agilent 7890B GC
Injection mode	Split (ratio = 30:1)
Injector temperature	350 °C
Carrier gas	Helium
Inlet pressure/total flow	116.48 kPa/44.88 mL min^−1^
Analytical column	UAMP column kit: UAMP-30M-0.5F (30 m × 0.25 mm × 0.5 µm, 5% diphenyl and 95% polydimethylsiloxane; and UAMP-2M-1.0F precolumn (2 m × 0.25 mm × 1.0 µm, 50% diphenyl and 50% polydimethylsiloxane) ^*a*^
Oven temperature program	40 °C isotherm (2 min), then to 280 °C at 20 °C/min and then hold for 10 min. Then to 300 °C at 40 °C/min (no hold, initiate backflush)
Transfer line temperature	280 °C
**3. Mass spectrometer**	
Instrument	Agilent 5977 MSD with extractor source
Mode	Scan
Scan range	*m/z* 29–350
Mode (detector voltage)	EI positive mode (70 eV)
Ion source temperature	230 °C
Quadrupole mass analyser temperature	150 °C
Pyrogram acquisition	MSD MassHunter (Agilent Technologies, USA)

^a^Supplied by Frontier Laboratories Ltd., Japan

The major adjustment to the previously reported method is the installation of a post-column backflush, which was programmed using the Agilent backflush wizard. For backflushing, the analytical column was connected to a purged union. A restrictor capillary connected the union to the MSD (inert fused silica capillary; 1.05 m × 150 µm, Agilent Technologies, USA). The post-run conditions were oven temperature of 320 °C, backflush duration of 6.06 min, 5.7 void volumes swept. Specifically, the backflush inlet pressure was 13.79 kPa and the auxiliary pressure at the purged union 294.81 kPa. This resulted in a column flow of −2.029 mL/min (i.e. towards the inlet) and a flow from the restrictor into the MSD of 10 mL/min. The method was retention time locked to styrene, which was regularly verified using 5 µg of polystyrene reference material.

The filters on which the MPs were recovered are produced from borosilicate glass fibres, and no diluent (SiO_2_ or CaCO_3_) was added to the sample discs for pyrolysis. For quantitation, characteristic pyrolytic products of the eleven common microplastic polymers were selected and calibrations were created using the MPs-SiO_2_ standard with silica as an inert diluent (0.4, 0.8, 1.2, 2.0 and 4.0 mg of the blended commercial standard). Pyrograms were inspected using F-Search All-in-One (version 3.7.0, Frontier Laboratories Ltd, Japan) and Agilent’s Enhanced Data Analysis. Mass spectral data of the MPs-SiO_2_ standard were deconvoluted in Unknowns Analysis and then subjected to library search using NIST 17 and NIST 23 spectral libraries, as well as an in-house library of the characteristic pyrolysates.

Quantification of MPs was performed using a custom method set up in Mass Hunter Quantitative Analysis for GC-MS. Responses obtained from integration of peak areas of the ions of the quantification pyrolysates were exported into MATLAB (version R2024b, MathWorks Inc., USA) where they were processed using a custom script that fitted the peak area against the mass of the calibration samples using a linear regression model. The residuals and the fitted calibration curves of the pyrolysates were plotted and checked using Lack-of-Fit *F*-test. The MATLAB script also calculated the limit of detection (LOD) and limit of quantification (LOQ) according to 3 times and 10 times, respectively, the standard deviation of repeatability divided by the calibration curves’ slope. The polymer concentrations in the samples were flagged as < LOD, < LOQ or valid before exporting the results.

### Quality control and quality assurance

Contamination of samples was prevented through the use of glass, aluminium foil and stainless steel/metallic materials. Cotton laboratory coats and exclusively nitrile gloves were worn to preclude plastic contamination of other materials such as analytical reagents, extracted MPs and equipment. To avoid cross-contamination, thorough cleaning of the scissors before and between dissections and the use of separate aluminium foil per fish was mandatory. Distilled water, potassium hydroxide and zinc chloride solutions used were pre-filtered through microfibre glass filters to prevent contamination.

Procedural blanks were prepared along with the samples to assess if any contamination arose from preparation and handling of the samples. Discs from filters analysed by Pyr-GC-MS were prepared in a flow hood and kept covered with aluminium foil while being transferred onto the auto-shot sampler. Contamination was checked through the use of empty eco-cups as blanks and pyrolysis of punched discs from unused filters and the nitrile gloves used in handling samples. The mass spectrometer detector was tuned daily before analysis to ensure reproducible resolution and sensitivity during analysis.

### Statistical data analysis

Normality of quantitative data was assessed using the Shapiro-Wilk test. Depending on the data distribution, two-way analysis of variance (ANOVA), Kruskal-Walli’s test or Mann-Whitney *U* test as appropriate was used to evaluate the effects of seasons and sampling sites on the morphometric data of fish, number of ingested plastic items, microplastic colours, forms, sizes and their interactions. Whenever statistically significant differences were detected, Tukey’s range test or Dunn’s post hoc test with Bonferroni correction was used to compare group means or mean ranks. Data visualisation and statistical tests were performed in Origin Pro 2026 software for Windows (OriginLab Corporation, Northampton, MA) at 95% confidence interval.

## Results and discussion

### Contamination control

During sample preparation, the procedural blanks (*n* = 14) had no detectable MPs, indicating the absence of a detectable contribution by contamination. Airborne MPs during microscopic analysis were also considered negligible since none of the control filters (*n* = 16) had detectable MPs. Empty eco-cups and nitrile gloves had no contribution to the responses of the pyrolysates used for quantitative analysis of MPs.

### Only gastric fish species show ingested microplastics

Initially, it was hypothesised that MPs uptake by the planktivorous species (*R. argentea*) would be higher owing to its opportunistic omnivory and diel vertical migratory behaviours. However, no MPs were detected in whole samples of this species in either season. Similarly, edible tissues of *S. victoriae* and *P. aethiopicus*, and the gills of *S. victoriae* did not contain detectable MPs. The total number of anthropogenic debris identified in the GIT of *P. aethiopicus* and *S. victoriae* was 314, that is 145 and 169 particles, respectively (Supplementary Table S2). Mean microplastic abundances ranged from 3 to 25 items/fish, with the lowest (5.0 ± 1.7 and 4.7 ± 0.6 (standard deviation) items/fish for *P. aethiopicus* and *S. victoriae*) and highest (16.0 ± 7.8 and 14.7 ± 6.7 items/fish) mean number of ingested MPs recorded in dry season samples from Port Bell and Ripon Falls, respectively.

Considering seasons, *P. aethiopicus* had slightly higher MPs load in the dry season (0.11 ± 0.04 per fish, 0.35 ± 0.27 items/g of GIT) compared to the wet season (0.16 ± 0.11 per fish, 0.30 ± 0.22 items/g of GIT), but this was not statistically significant as per Mann-Whitney *U* test (*U* = 45.5, *P* = 0.68). The average number of MPs increased in fish sampled from Katosi and Port Bell during the wet season when compared to the dry season. However, Kruskal-Walli’s test indicated that sampling location did not significantly influence the number of MPs ingested by *P. aethiopicus* (*H* = 6.4, *P* = 0.27). In contrast, the MPs load in *S. victoriae* were lower in the dry season (1.04 ± 0.17 items per fish, 8.49 ± 0.50 items/g of GIT) than in the wet season samples (1.27 ± 0.69 items per fish, 8.63 ± 2.38 items/g of GIT). Kruskal-Walli’s test indicated significant differences in the number of MPs ingested by *S. victoriae* from the three FLBs (*H* = 14.5, adjusted *P* = 0.01).

Kruskal-Walli’s test across combined groups defined by species, FLBs and seasons confirmed that there was a relatively higher MPs load in *S. victoriae* than in *P. aethiopicus* (*H* = 31.7, adjusted *P* = 0.01). Post hoc pairwise comparisons using Dunn’s test indicated a significant difference in MPs load between dry season samples of *P. aethiopicus* from Port Bell and wet season samples of *S. victoriae* from Katosi (adjusted *P* = 0.04; see Supplementary Table S2). *S. victoriae* sampled from Port Bell during dry season had marginally lower MPs load than from Katosi, although this was not statistically significant after adjustment for multiple comparisons (adjusted *P* = 0.057).

The non-detection of MPs in whole *R. argentea* samples in the present study may be explained by the fact that it is agastric, and therefore a longer retention of ingested MPs is less likely than in gastric fish species. Furthermore, cyprinids rely on visual cues while foraging and possess well-developed chemosenses based on environmental needs, which enable them to assess the edibility and decide whether or not to swallow food items, reducing the chances of inadvertent MPs ingestion (Gebhardt & Hofmann, 2023). It is to be emphasised that feeding mode and species-level anatomical specialisations play an important role in the selection behaviour related to MPs ingestion and retention in fish (Kumkar et al., 2021). Although pelagic vertical migratory fish species like *R. argentea* may contribute to the redistribution of MPs within the water column, this does not necessarily increase ingestion if the fish feed selectively. That notwithstanding, it should be noted that the present study only considered MPs larger than 0.25 mm in their longest or widest dimension. Furthermore, these fish were analysed whole, which may dilute MPs associated with the intestinal bulb and respiratory structures relative to the total sample mass.

The gills of *S. victoriae* had no detectable MPs, indicating that direct oral intake is the primary MPs exposure route in this species (Valente et al., 2023). The high MPs ingestion frequencies observed in fish from Ripon Falls indicates a possible relationship between microplastic contamination, and the proximity of recreational beaches and the slum settlement with poor waste management to this FLB (Egessa et al., 2020). Marbled lungfish (*P. aethiopicus*) is the longest extant lungfish, and its lower MPs load than the upside-down catfish (*S. victoriae*) leads to the reasoning that other traits rather than its body size (that is, length used here as a proxy for fish age; Supplementary Table S3) influence its MPs ingestion dynamics. The higher MPs load in *S. victoriae* may be attributed to its greater interaction with suspended MPs during benthopelagic foraging compared to *P. aethiopicus*, a demersal species that aestivates during the dry season and under anoxic conditions (Goudswaard et al., 2002). Overall, the present results align with recent conclusions that MPs ingestion by wild fish is not a function of its body size and gut fullness (Atukwatse et al., 2026; de Vries et al., 2020; Güven et al., 2017).

The sampled fish species are trophically linked (Supplementary Table S1). In the present study, no incidental observations of biomagnification or trophic transfer were made. In *R. argentea*, no MPs > 0.25 mm were found. Therefore, a formal assessment of potential MPs transfer from this species to its predators is precluded. In *S. victoriae*, the recorded MPs load was higher than in *P. aethiopicus*, which is at a higher trophic level. The present findings therefore do not provide direct evidence of possible trophic transfer or biomagnification of MPs. Nevertheless, these phenomena cannot be ruled out, especially under conditions of chronic exposure to MPs < 0.25 mm in sizes, yet their confirmation or conclusive rejection would have to be based on a dedicated study design.

According to previous studies on L. Victoria, MPs were detected in the GIT of *S. victoriae* (1–2.6 items/fish), *O. niloticus* (1–81 items/fish), *C. gariepinus* (1–4 items/fish) and* L. niloticus* (2–4.3 items/fish) (Atukwatse et al., 2026; Gathu et al., 2024; Mutesi, 2023; Nicholson, 2021). The presence of MPs in a species of the *Protopterus* genus is reported here for the first time. However, the occurrence of plastic debris in the GIT of freshwater fishes destined for human consumption has been reported globally. For example, Merga et al. (2020) found 1–26 items/fish in *O. niloticus* and *C. gariepinus* from Lake Ziway, Ethiopia, while fish from Lake Titicaca and Tocagua Lake (Colombia) contained up to 20 items/GIT (Loayza et al., 2022; Miranda-Peña et al., 2023). The abundance of plastic items in fish in the present study is lower than some of the highest mean microplastic levels such as 59 and 138 items/GIT in fish from Lake Ontario and Lake Superior (USA) (Milne et al., 2024; Munno et al., 2022).

### Blue and brown MPs are predominant in *P. aethiopicus* and *S. victoriae*

**Fig. 1:**
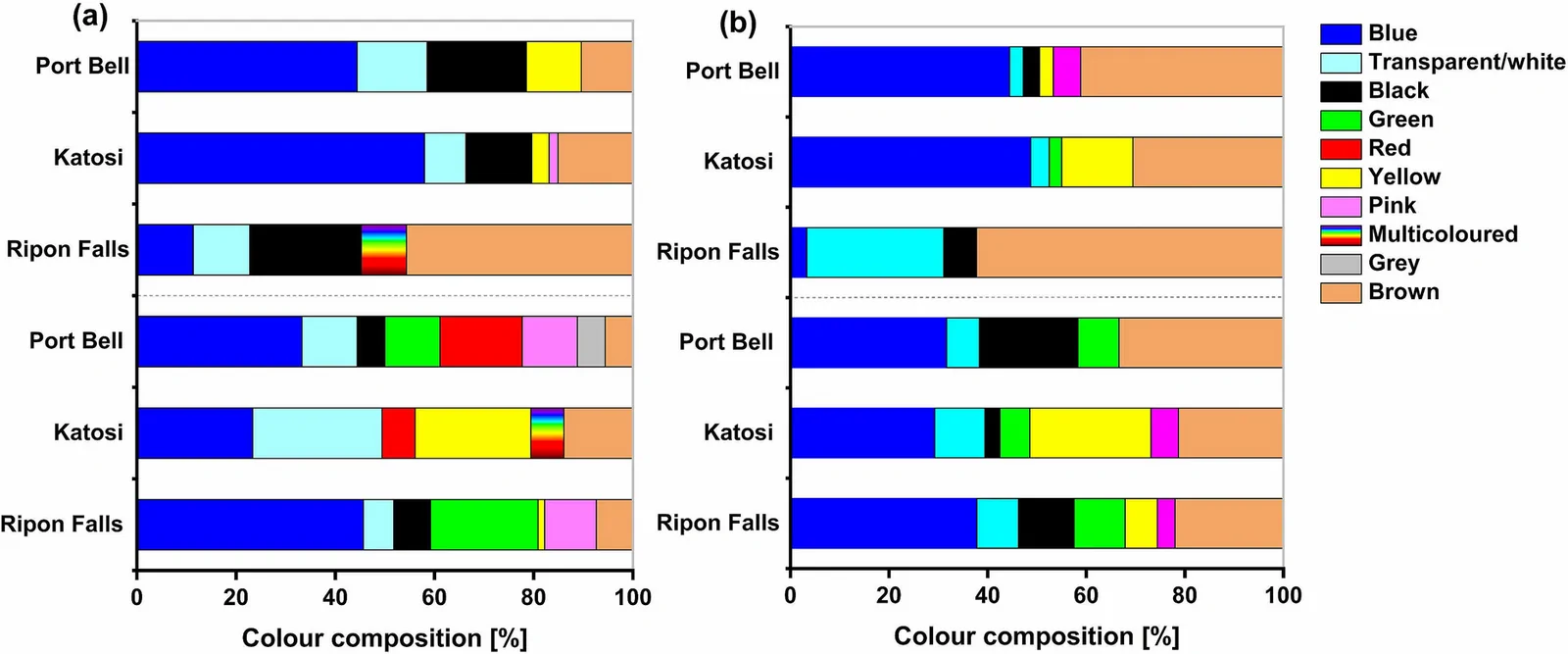
Proportion of microplastic colours identified in **a** *P. aethiopicus* and **b** *S. victoriae*. The left and right panel in each case represent samples collected during the wet and dry seasons, respectively

Ingestion of MPs by colour showed intra- and interspecific variations (Fig. 1). Blue was the colour of most items (45.7% and 33.3%) in *P. aethiopicus* sampled from Ripon Falls and Port Bell during the dry season. At Katosi, transparent items dominated (26.1%), followed by those that were blue and yellow (23.3% each). In the wet season, blue plastics were the dominant items (58.0% and 44.4%) in samples from Katosi and Port Bell, whereas brown (45.7%) and black (22.5%) items were prevalent in samples from Ripon Falls. These colour variations across FLBs and between seasons were not statistically significant (*H* = 38.6, *P* = 0.27).

Ingested MPs in *S. victoriae* were mainly blue (29.3–48.8%) or brown (21.3–62.2%), except for fish sampled from Ripon Falls during the wet season where blue MPs accounted for only 3.3% of the total ingested plastic debris. Unlike in *P. aethiopicus*, where ten different colours of MPs were identified, grey, multicoloured and red particles were not detected in the gut contents of *S. victoriae*. The distribution of MPs colour ingested by *S. victoriae* was not significantly different among FLBs and between seasons (*H* = 46.8, *P* = 0.06). Kruskal-Walli’s test across combined groups defined by species, FLBs and seasons indicated that there was no significant difference among the colours of plastic items ingested by *P. aethiopicus* and *S. victoriae* (*H* = 87.0, *P* = 0.07).

The multicoloured particles observed in *P. aethiopicus* may result from manufacturing of the plastic material, which might involve the application of multiple pigment layers, or partial weathering that exposes underlying colours. In L. Victoria fish, Gathu et al. (2024) and Atukwatse et al. (2026) observed mostly clear and blue MPs, with red, black and green coloured particles occurring at lower proportions. The other studies on MPs ingested by fish species from L. Victoria (Mutesi, 2023; Nicholson, 2021) did not report particle colours. The diversity of MP colours in the present study agrees with previous studies which cited the dominance of blue MPs in fish from Lake Ziway (Merga et al., 2020), Lake Titicaca (Loayza et al., 2022), Lake Ontario and Lake Superior (Munno et al., 2022).

Fish often ingest blue plastic items because blue is the most common and photostable colour used in synthetic materials (Christie & Abel, 2021). In Uganda, barrels and plastic caps of mineral water bottles are predominantly blue, and these bottles are usually repurposed as floats for fishing nets in L. Victoria (Egessa et al., 2020). Fishermen also use pieces of blue plastic strand slippers because they are easy to recognise, making identification and retrieval of fish nets easy. These floats were observed during trawling of L. Victoria surface waters, and under strong hydrodynamic stress, they can fragment into MPs that are later ingested by fish. Brown MPs may result from prolonged exposure to ultraviolet radiation, oxidation and biofouling, all of which cause discolouration and weathering-induced browning (i.e. the formation of chromophores from the oxidation of polymers, or quinonoid structures from the oxidation of phenolic antioxidants) (Zhao et al., 2022).

### Fragments and filaments were the commonly ingested MP forms

In the dry season, fragments (71.0%) followed by filaments (16.4%) and fibres (9.4%) were the most abundant in *P. aethiopicus* (Supplementary Table S4). Samples from Ripon Falls had the highest proportion of fragments (77.1%) while the least was in samples from Port Bell (55.6%). Pellets (0.9%) and foam (2.2%) were also recorded in samples from Ripon Falls and Katosi, respectively. A similar ingestion frequency to that in *P. aethiopicus* was observed in *S. victoriae*, giving a trend of fragments (70.9%) > filaments (15.8%) > fibres (7.4%) > films (5.9%).

**Fig. 2:**
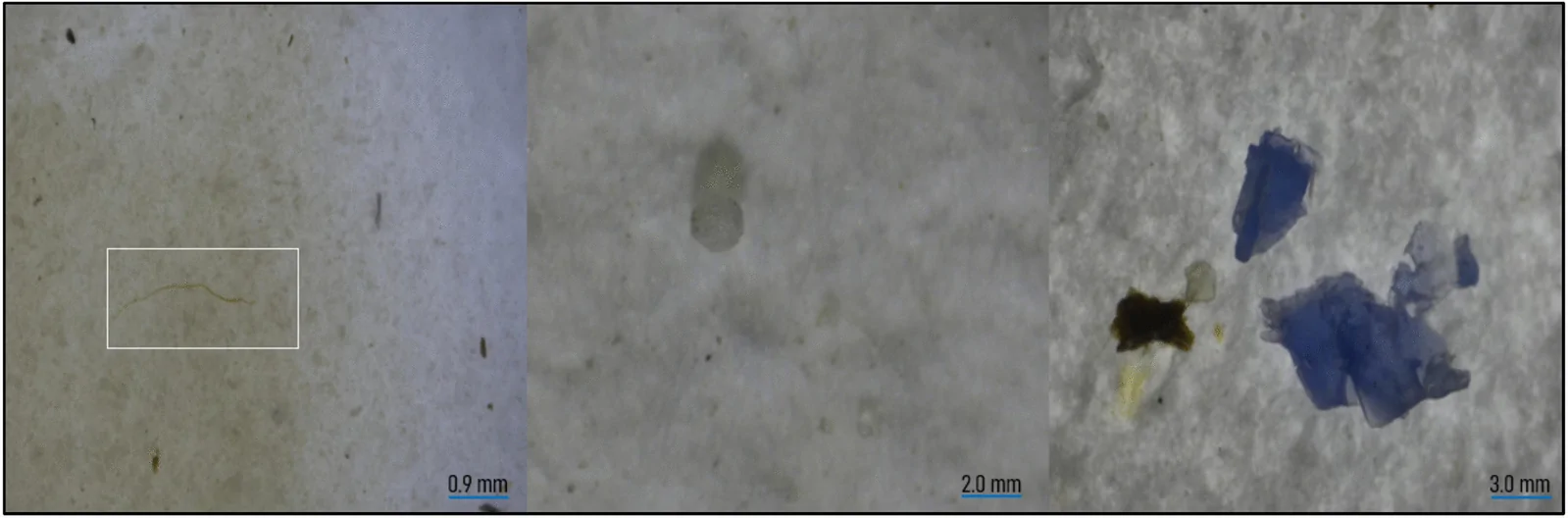
Morphology of plastic items observed in the gastrointestinal tracts of *P. aethiopicus* and *S. victoriae*. From left to right: fibre, pellet and fragments

In the wet season samples, the trend was fragments (61.5%) > filaments (25.0%) > fibres (10.8%) > films (2.8%) in *P. aethiopicus*, and fragments (71.9%) > filaments (14.9%) > fibres (9.4%) > films (3.7%) in *S. victoriae*. According to Kruskal-Walli’s test, there were significant differences among the particle morphologies observed in *P. aethiopicus* (*H* = 86.7, adjusted *P* < 0.01), *S. victoriae* (*H* = 51.8, adjusted *P* = 0.04) and between species (*H* = 138.4, adjusted *P* < 0.01). *Protopterus aethiopicus* at a higher trophic level ingested a greater diversity of microplastic forms. These included a pellet (Fig. 2) and a foam which were not encountered in *S. victoriae*. Such a tendency may be due to its larger gape or lower selectivity for prey items, thereby encouraging inadvertent ingestion of MPs (Valente et al., 2023).

Previous studies concluded that fibres, filaments, fragments, pellets, films, foam and beads were the forms of MPs in fish sampled from L. Victoria (Atukwatse et al., 2026; Gathu et al., 2024; Mutesi, 2023; Nicholson, 2021). Similarly, fish from Lake Ziway (Merga et al., 2020), Lake Ontario, Lake Superior (Munno et al., 2022), Lake Titicaca (Loayza et al., 2022) and Tocagua Lake (Miranda-Peña et al., 2023) contained mainly fibres and fragments in their GIT, which are similar to the results obtained in the current study. The dominance of fragments in the GIT of *P. aethiopicus* and *S. victoriae* is expected because they occupy demersal and benthopelagic zones, which exposes them to higher concentrations of sunk and settled plastic fragments (Parker et al., 2021).

### Microparticles ingested were mostly small-sized debris (0.3–1.9 mm)

Ingested plastic debris were grouped into five size classes. Particles measuring 0.3–0.9 mm and 1.0–1.9 mm constituted more than 80% of the detected items in *P. aethiopicus* during the dry season. In the wet season, the two size groups contributed greater than 55% of the identified MPs (Supplementary Fig. S1). Plastics measuring 4.0–4.9 mm were not recovered in any of the *P. aethiopicus* samples, while those in the size range of 2.0–2.9 mm and 3.0–3.9 mm together contributed 8–20% and 9.0–41.6% of the total plastics detected in the dry and wet seasons, respectively. Fish from Ripon Falls had the highest levels (91.7% and 69.7%) of small-sized plastics (0.3–0.9 mm) in the two seasons. At Katosi and Port Bell, this trend was obscure, with samples from the latter having a higher level of small-sized debris during the dry season. These spatiotemporal variations were not statistically significant (*H* = 32.0, *P* = 0.10).

In *S. victoriae*, MPs in the size range of 0.3–0.9 mm (42.7–80.0%) were the most common plastic size categories among the FLBs and between seasons (Supplementary Fig. S2). In both seasons, samples from Port Bell had the highest percentage (80.0% and 68.9%) of 0.3–0.9-mm particles, similar to the trend observed at Ripon Falls for *P. aethiopicus*. At Ripon Falls, 1.0–1.9-mm MPs (19.6% and 24.4%) was the second most dominant size group. Interestingly, *S. victoriae* samples from Katosi and Port Bell had 3.0–3.9-mm plastics (20.0–38.5%) as the second most prevalent size group. Unlike in *P. aethiopicus*, MPs measuring 4.0–4.9 mm were identified in *S. victoriae*, but they contributed only 6.5–8.6% of the plastic debris identified. The variations in the size groups were found to be statistically different among the FLBs and between seasons (*H* = 43.8, adjusted *P* = 0.04). Interspecies comparison showed that the spatiotemporal variations in microplastic sizes were significant (*H* = 76.2, adjusted *P* = 0.02).

Gape size can influence the upper size limit of ingestible MPs, but the absence of 4.0–4.9-mm MPs in *P. aethiopicus* (with a wider gape than *S. victoriae*, and in which larger MPs were detected) could thus be related to differences in habitat exposure, environmental availability of larger MP particles, selective rejection of non-food items or egestion dynamics rather than gape size limitation. Only one earlier study in L. Victoria determined the sizes of MPs in fish GIT to range between 0.07 and 1.5 mm (Gathu et al., 2024). Fish ingest MPs of various sizes, but smaller particles (< 1 mm) have been reported at higher proportions (Gathu et al., 2024; Saad & Alamin, 2023). This may be attributed to passive uptake and the tendency of fish to actively avoid larger MPs. Furthermore, fish may fragment ingested MPs into smaller debris through the mechanical action of chewing, chemical and biological digestion (Parker et al., 2021).

### Nylons dominated the polymer types according to µ-FTIR

In the sample subset subjected to µ-FTIR analysis, 34.8% (32/92) of the particles were confirmed to be synthetic plastic polymers. In *P. aethiopicus*, the MPs comprised five polymer types, with nylon 6/66 copolymer (52.9%) as the most common (Supplementary Table S5). Nylon (amorphous; 17.6%), polyethylene (PE; 11.8%), polypropylene (PP; 11.8%) and nylon 6/10 copolymer (5.9%) were detected at lower proportions in the samples. Spatiotemporally, nylon 6/66 (33.3%), nylon (33.3%) and PE (22.2%) were the dominant polymer types in the dry season. In the wet season, nylon 6/66 (75%) was the dominant polymer, followed by PP and nylon 6/10 (12.5% each).

**Fig. 3:**
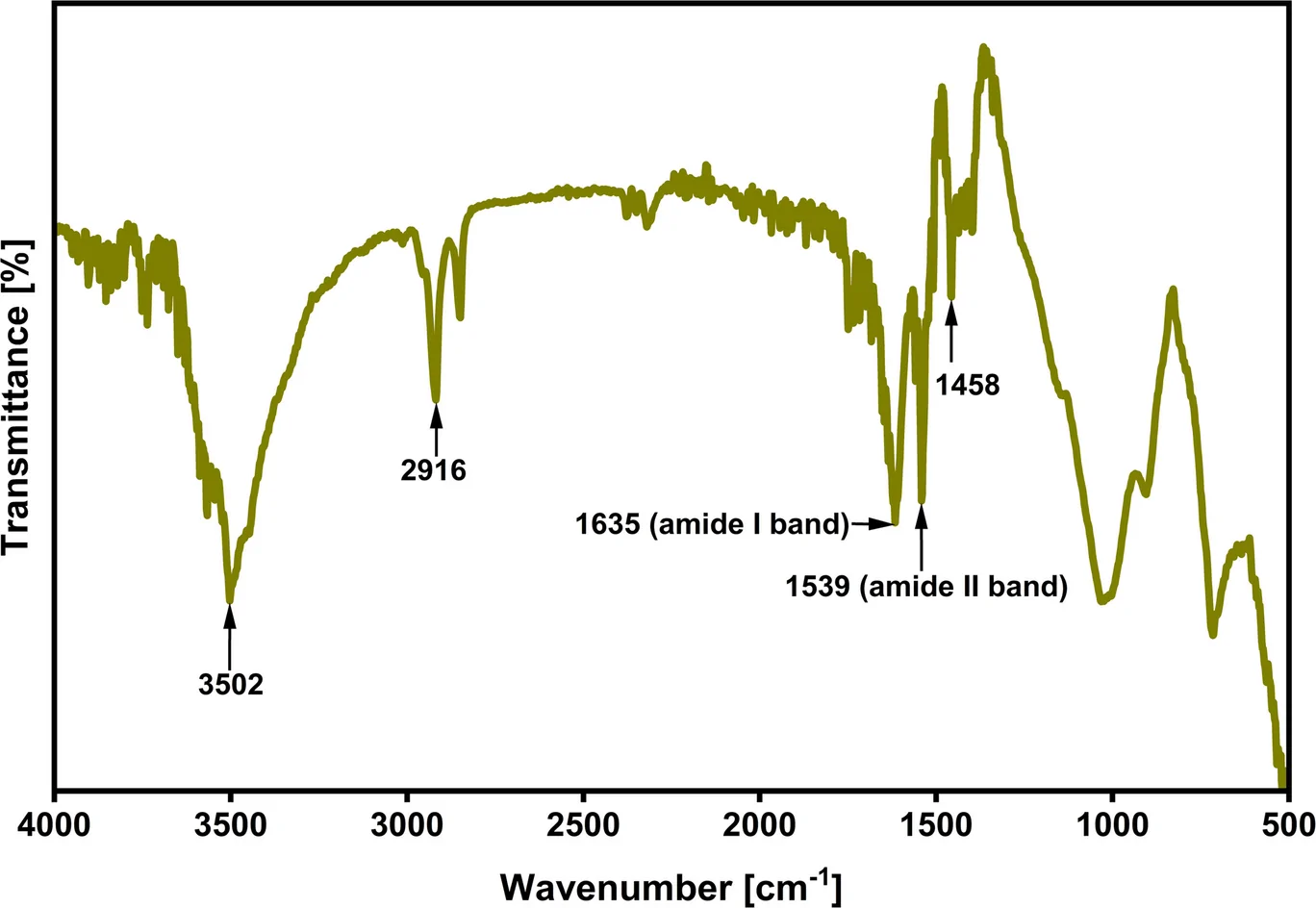
µ-FTIR spectrum of a nylon 11 particle identified in the gastrointestinal tract of *S. victoriae.* Arrows indicate the wavenumbers of prominent bands

*Synodontis victoriae* had five polymers in the order: nylon 11 (50%) > nylon (18.8%) > PE (12.5%) = nylon 66 > PP (6.2%). By sampling site, nylon 66 (50%) and nylon 11 (50%) were confirmed in samples from Ripon Falls during the dry season while the wet season samples contained nylon 11 (66.7%) and PP (33.3%). At Katosi, nylon 11 and PE (100% each) were the polymers identified during the dry and wet seasons, respectively. For Port Bell samples, nylon (amorphous), nylon 66 and nylon 11 (33.3% each) were the plastic polymers confirmed in dry season samples while wet season samples had solely nylon. Seasonwise, nylon 11 (33.3%), PE (33.3%), nylon (16.7%) and nylon 66 (16.7%) were the polymers identified in *S. victoriae* sampled during the dry season while nylon 11 (66.7%; Fig. 3), nylon (22.2%) and PP (11.1%) were identified in wet season samples.

Recently, polystyrene, PE, PP and poly(perfluorobutadiene) were identified as the plastic polymers in the GIT of fish from L. Victoria (Atukwatse et al., 2026; Gathu et al., 2024). Regarding the dominance of nylons in the GIT of fish in the present study, it could plausibly be linked to the nylon gillnets and longlines used in L. Victoria fisheries (Egessa et al., 2020; Nicholson, 2021), and the higher density and gut retention time of these polymers compared to polyolefins. Relevant to this discussion also is the distinct FTIR signatures of nylons (that is, C=O stretch around 1630–1690 cm^−1^, NH bend around 1530–1550 cm^−1^ and NH stretch between 3200 and 3500 cm^−1^) which eases their detection in complex matrices compared to PE and PP (Veerasingam et al., 2020). The spectral fingerprint of a nylon 11 particle identified in *S. victoriae*, for instance, featured wavenumbers characteristic of amide group NH stretching (3502 cm^−1^), aliphatic CH stretching (2916 cm^−1^), C=O stretching (1635 cm^−1^; amide I band), bending vibrations of NH bond and stretching vibrations of CN bonds (1539 cm^−1^; amide II band), and CH_2_ bending vibration (1458 cm^−1^; Fig. 3). Conversely, the FTIR spectrum of a representative PP microparticle isolated from *P. aethiopicus* GIT (Supplementary Fig. S3) had CH stretching (2839 and 2918 cm^−1^), CH_2_ bending (1456 cm^−1^), CH_3_ bending (1375 cm^−1^), CH_3_ rocking (1166 cm^−1^) and CC stretching vibrations (997 cm^−1^) consistent with the published prominent FTIR wavenumbers of this polyolefin (Veerasingam et al., 2020).

### Post column backflush enhances Pyr-GC-MS performance

In our earlier work on eutrophic surface water, higher boiling point compounds fouled the electron ionisation source, reduced detector sensitivity and necessitated weekly ion source cleaning. Tuning of the MSD by perfluorotributylamine and replacement of quartz pyrolysis tubes and of inlet septa were also more frequently required to ensure reliable performance of the Pyr-GC-MS system. The samples in the present study were even more challenging due to the lipid-rich nature of the fish tissues. To increase stability and thus reliability of the MPs analysis, several measures were taken: a post-column backflush was installed, the retention time was locked to styrene and two sets of standards, MPs-SiO_2_, MPs-CaCO_3_ and their mixture, were used to identify the last peaks of interest (Supplementary Fig. S4 and Fig. S5). In the MPs-SiO_2_ standard, the commonly targeted styrene trimer (2,4,6-triphenyl-1-hexene) was detected only at the rather low 0.8 mg calibration level; therefore, the quantification of polystyrene was performed using styrene dimer (1,3-diphenylbut-3-ene). The chromatographic run was ended after the last peak of interest had eluted (at 24.475 min), and a post-run backflush was initiated to clean the column and inlet within 6.06 min. This adjustment shortened the sample analysis time by a fourth when compared to the original 40-min method, and prevented high-boiling, late eluting compounds from fouling the ion source and contaminating the analytical column. This also reduced baseline instability and retention time shifts.

Retention time locking to styrene allowed for refined MPs quantification utilising the MPs-SiO_2_ calibration previously used for L. Victoria surface waters. The quantifier and qualifier compounds of the eleven target polymers are shown in Table 2. The fitted external calibration curves for all the target polymers corresponded to the full calibration range (0.35–193.14 µg; R^2^ ≥ 0.975), with the calculated LOD and LOQ values of 0.01–14.71 µg and 0.03–49.06 µg, respectively (Omara et al., 2026).

**Table 2:** Diagnostic pyrolysates used for the analysis of microplastics in the gastrointestinal tracts of *P. aethiopicus* and *S. victoriae*

Microplastic polymers	Diagnostic pyrolysate(s)^a^	Pyrolysate abbreviation	Quant ion (main *m/z*)^c^	Qualifier ion (sub *m/z*)	*t*_R_ (min)
Nylon 66 (N66)	*Cyclopentanone*	CP	84	55, 56	8.808
Nylon 6/Polycaprolactam (N6)	*ε-Caprolactam*	Capro	85	63, 113	14.451
Poly(ethylene terephthalate) (PET)	*Benzoic acid*	BA	105	77, 122	12.910
	Benzophenone	BP	105	77, 182	17.368
Styrene-butadiene rubber (SBR)	*4-Phenylcyclohexene*	4-PCH	104	91, 158	14.626
	4-Vinylcyclohexene	4-VCH	108	54, 79	8.946
Polypropylene (PP)	2,3,3-Trimethyl-4-nonene	C12’	55	111, 168	20.568
	*2,4-Dimethyl-1-heptene*	C9’	55	70, 126	8.908
Polyethylene (PE)	1-Undecene	C11’	55	97, 111, 154	11.622
	*1,20-Heneicosadiene*	C21”	82	95, 292	20.948
Poly(methyl methacrylate) (PMMA)	*Methyl methacrylate*	MMA	69	99, 100	7.137
Polystyrene (PS)	α-Methyl styrene	AMS	118	103, 117	11.009
	*1,3-Diphenylbut-3-ene* (*styrene dimer*)	SS	91	208, 130	17.805
	Styrene (monomer) ^b^	S	104	77, 78	10.098
Acrylonitrile butadiene styrene terpolymer (ABS)	*2-Phenethyl-4-phenylpent-4-enenitrile*	SAS	170	91, 115	23.011
	3-Phenylpent-2-enenitrile	–	91	77, 157	15.564
	4-Phenylbutanenitrile	–	91	77, 145	14.866
Polycarbonate (PC)	4-Isopropenylphenol	IPP	134	91, 119	14.222
	*Bisphenol A*	BPA	213	119, 228	23.237
Poly(vinyl chloride) (PVC)	1,2-Dihydronaphthalene	–	115	129, 130	13.192
	*Naphthalene*	Nap	128	63, 115	13.489

^a^The *italicised* pyrolysate of each polymer was used for quantification

^b^Retention time (*t*_R_) locking pyrolysate. All other pyrolysate retention times were locked to styrene

^c^Primary quantitation ion chosen based on its intensity

### Refined quantitative Pyr-GC-MS confirmed and complemented µ-FTIR results

**Fig. 4:**
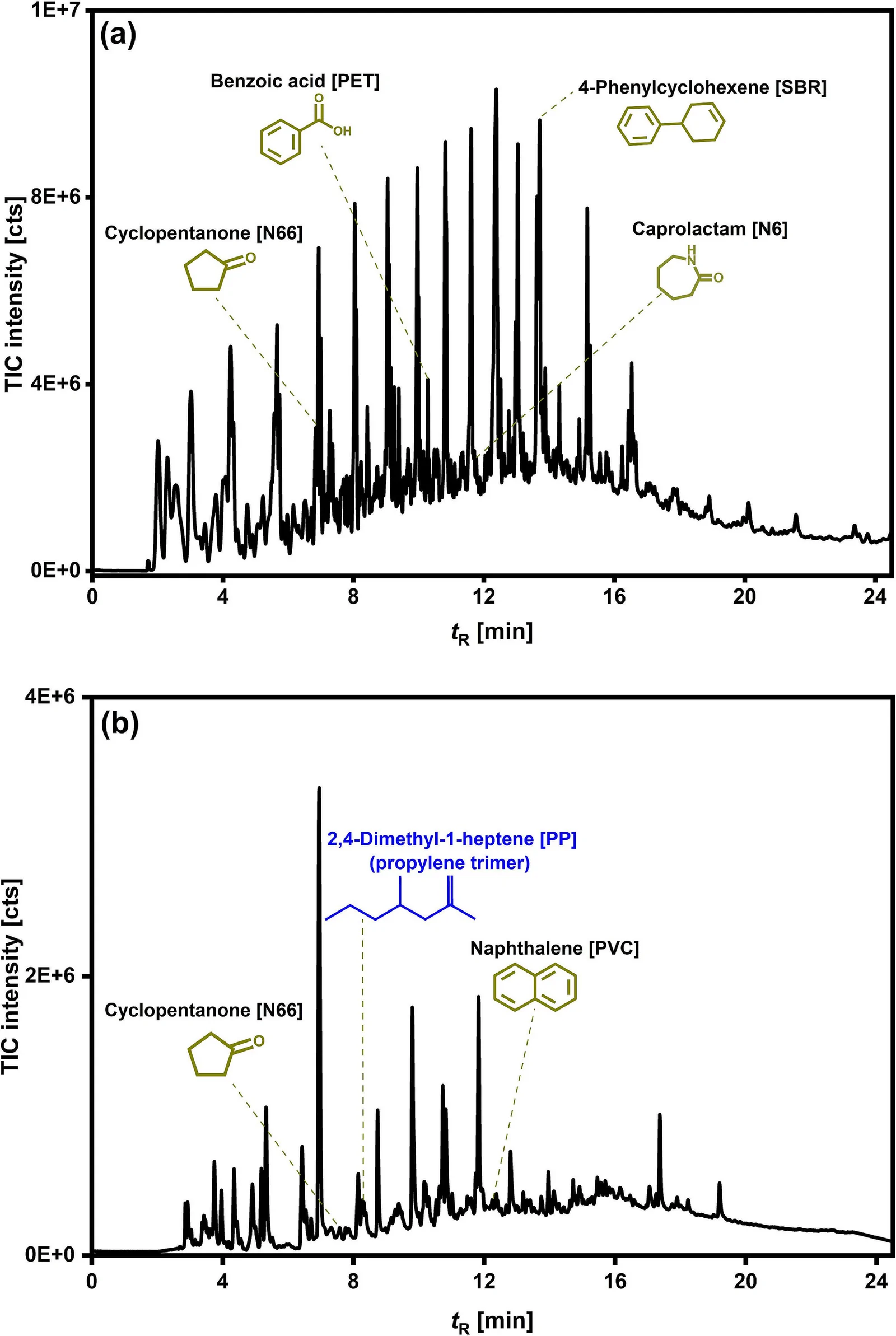
**a** Pyrogram of the GIT of *S. victoriae*. This sample had the highest number of quantified polymers. **b** Pyrogram of the GIT of *P. aethiopicus*. Here, polypropylene is above the limit of quantification

The refined Pyr-GC-MS method detected seven polymers above their respective LODs (Table 3; Fig. 4). Nylon 66 (243 µg/g, only in *S. victoriae*), nylon 6 (0.24–96 µg/g), PP (6.41 µg/g, only in *P. aethiopicus*), styrene-butadiene rubber (SBR; 8.5–34.8 µg/g) and poly(ethylene terephthalate) (PET; 1.23–107 µg/g) were quantified. The detection of nylon 6, nylon 66 and PP by Pyr-GC-MS, to a greater extent, confirmed the dominance of the nylons in the GIT and complemented µ-FTIR results. Other than in the wet season samples of *P. aethiopicus* from Port Bell, nylon 6 was detected above its LOD of 0.15 µg in both seasons. These nylon MPs could derive from the degradation of ghost nylon fishnets, lines and ropes used in L. Victoria (Egessa et al., 2020; Nicholson, 2021).

**Table 3:** Concentration of microplastic polymers (µg/g) in the gastrointestinal tracts of *P. aethiopicus* and *S. victoriae* from L. Victoria

Fish species	Polymer ^*a*^	Dry season [August 2023]^*b*^						Wet season [November 2023]					
		Ripon Falls 01	Ripon Falls 02	Katosi 01	Katosi 02	Port Bell 01	Port Bell 02	Ripon Falls 03	Ripon Falls 04	Katosi 03	Katosi 04	Port Bell 03	Port Bell 04
*P. aethiopicus*	N66	-	-	-	-	-	-	-	-	-	-	< *LOQ*	-
	N6	1.09	0.24	< *LOQ*	< *LOQ*	0.29	< *LOQ*	< *LOQ*	1.26	< *LOQ*	1.02	-	0.63
	PET	1.23	2.54	1.67	-	3.25	4.47	2.87	3.61	2.15	1.98	3.26	3.41
	SBR	-	-	-	-	-	-	-	-	-	-	8.50	-
	PP	-	-	-	-	6.41	-	-	-	-	-	-	-
*S. victoriae*	N66	243	-	-	-	-	-	< *LOQ*	-	-	< *LOQ*	< *LOQ*	< *LOQ*
	N6	53.4	< *LOQ*	1.97	< *LOQ*	< *LOQ*	20.8	14.1	4.56	< *LOQ*	96.2	29.9	72.0
	PET	23.5	21.5	20.6	19.8	48.3	74.4	107	74.4	19.3	22.0	26.3	41.8
	SBR	23.0	-	-	-	-	< *LOQ*	-	-	-	-	34.8	< *LOQ*
	PP	-	-	-	-	-	-	-	-	-	-	-	< *LOQ*
	PE	-	-	-	-	-	-	-	-	< *LOQ*	-	-	-
	PMMA	-	-	-	-	-	-	-	-	-	-	-	< *LOQ*

^a^Concentrations are normalised to the whole filter and mass of the gastrointestinal tracts. Polymer abbreviations follow those in Table 2

^b^ - indicates not detected (lower than the limit of detection), *LOQ* limit of quantification

Nylon 66 was detected at the highest concentration and only in samples from Ripon Falls and Port Bell. This may result from the extensive use of old car tires as cobbles near the Ripon’s pier and the lake shores (Supplementary Fig. S6), as well as the heavy freight truck traffic at Port Bell. Nylon 66 is a major component of tire cord fabric, and the quantification of SBR at only these FLBs further supports the apportioned sources. Car tire treads are composed of SBR as the main elastomer (30–70%), and as they undergo abrasion against road surfaces, SBR tire and road wear particles are released into the environment (Miera-Domínguez et al., 2024). Similar to nylon 6, PET was detected in all but one sample of *P. aethiopicus* from Katosi. µ-FTIR did not reliably identify any MPs composed of PET, which may be due to matrix effects, weathering or pigmentation that can obscure spectral features of MPs. In the current study, particles with spectral matches scoring < 60% in polymer libraries were excluded. Furthermore, subsampling bias can also lead to missing of PET particles, since µ-FTIR was performed on visually selected particles. Nevertheless, Pyr-GC-MS is a more robust and superior technique for unambiguous identification of PET when compared to µ-FTIR. PET is the fourth most produced polymer after PE, PP and poly(vinyl chloride) (PVC), and a major component of peanut jars, mineral water and soft drink bottles in Uganda (see Supplementary Fig. S6) (Wandeka et al., 2022).

**Fig. 5:**
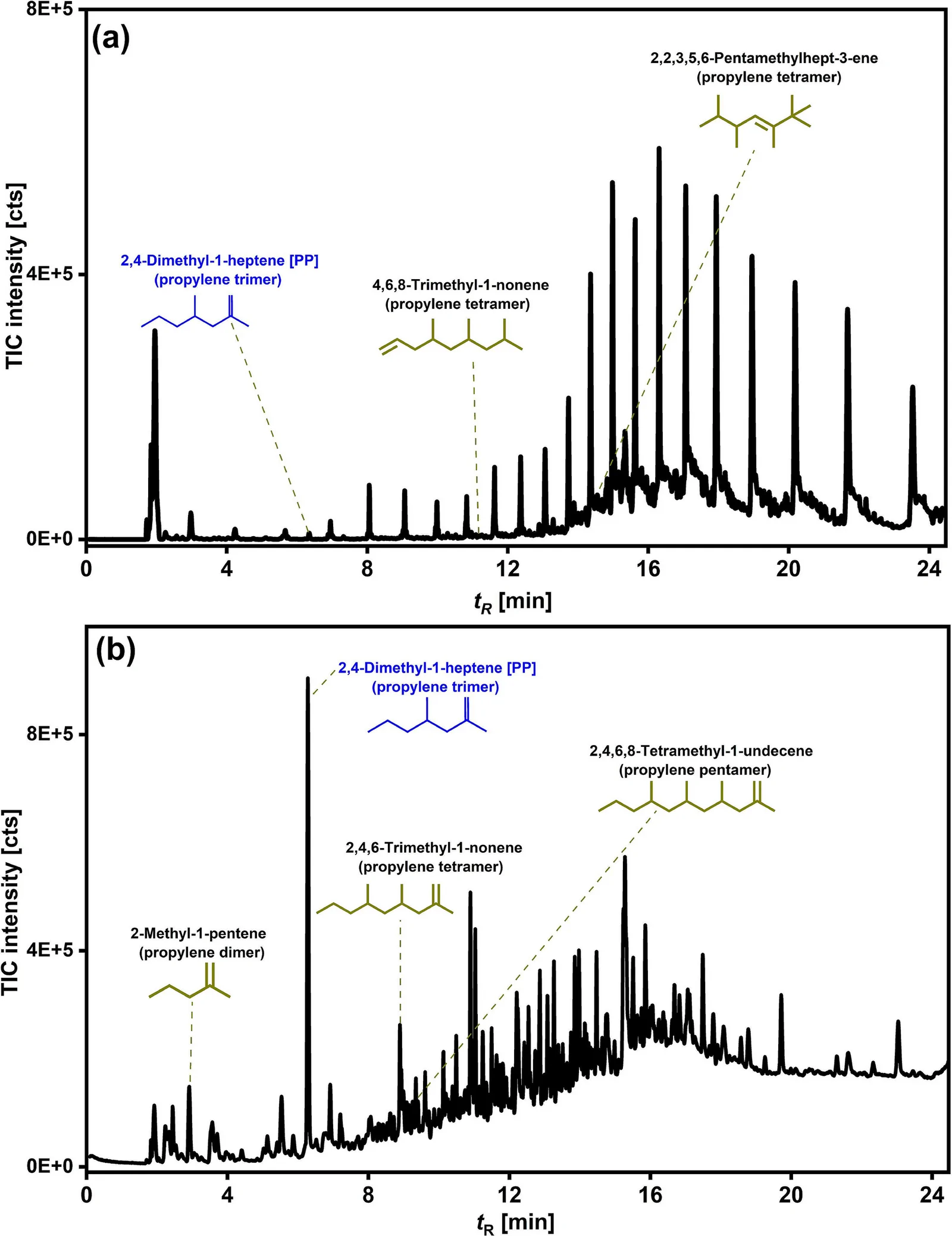
Pyrograms of propylene oligomer clusters and other canonical markers of polypropylene traced in the gastrointestinal tract of **a ***P. aethiopicus* and **b ***S. victoriae*

Polypropylene was detected above its LOD of 4.24 µg in two samples (*P. aethiopicus* and *S. victoriae* from Port Bell), although it was only quantifiable in the former (Fig. 4). As per µ-FTIR results, three particles were composed of isotactic PP (Supplementary Table S5). The non-detection of PP in most samples by Pyr-GC-MS could be rationalised by the presence of methyl substituents on the polymer backbone, which is known to promote more diverse backbiting reactions than in other polyolefins (Kruse et al., 2003). The polymer is therefore degraded into a larger variety of products during pyrolysis, thus effectively diluting the signal intensity over a large number of peaks. In addition, these reactions are influenced by stereoregularity (tacticity) and photooxidation of environmental MPs, which affects crystallinity and in turn the thermal degradation onset temperature and fragment distribution (Brits et al., 2024; Martínez et al., 2017; Toapanta et al., 2021). The relative proportions of the pyrolysis products may therefore vary. The GIT pyrograms in the present study were found to have various propylene oligomers (see Fig. 4 and Fig. 5; Supplementary Table S6). These propylene oligomers are unlikely to originate from matrix only, as the pyrograms of (relatively clean) water samples from L. Victoria and a PP 3D printing filament showed the same patterns of propylene oligomers (Supplementary Fig. S7). On account of the fact that such pyrograms with a large fraction of higher propylene oligomers showed only undetectable or traces of the trimer targeted for quantification, PP would be considered undetected in fourteen GIT samples, even though the PP signature is evident. A contrasting interference was noted in the literature where oligomers of ethylene terephthalate led to falsely increased concentrations of PET (Jeffries et al., 2026) instead of false negatives observed for PP in the present study.

Considering PVC, traces of naphthalene and its derivatives (1,2-hydronaphthalene, 2-methylnaphthalene, 1,4-dimethylnaphthalene and 1,4,6-trimethylnaphthalene) were present in the GIT pyrograms (Fig. 4; Supplementary Table S6). Based on previous recommendations, PVC is considered “detected” if the primary marker (naphthalene) is present with benzene in comparable ratios (up to a maximum of twofold) (Dehaut et al., 2020; Jeffries et al., 2025). In general, PVC is one of the most challenging microplastic polymers to quantify by Pyr-GC-MS, and some studies excluded it from their target analytes (Albignac et al., 2022; Jeffries et al., 2025; Sefiloglu et al., 2024) or relied on pyrolytically abundant but less specific naphthalene derivatives (Okoffo & Thomas, 2024; Peel et al., 2025), benzene (Ribeiro et al., 2020) or indene (Hermabessiere et al., 2023). Poly(methyl methacrylate) (PMMA) and PE were detected in wet season samples of *S. victoriae* from Port Bell and Katosi (Table 3). This aligns with our previous detection and quantification of PMMA (0.0036 µg/L) and PE (0.058–0.34 µg/L) in surface water from the same FLBs (Omara et al., 2026). For PMMA, the postulation was that it could come from the abrasion of structural plastics, protective coatings and paints since there are active shipping lanes at Port Bell (Dibke et al., 2021). The other polymers (acrylonitrile butadiene styrene terpolymer, polycarbonate and polystyrene) were not detected in any of the samples.

Few studies in published literature performed simultaneous Pyr-GC-MS quantification of all the eleven target microplastic polymers in wild fish tissues as investigated in the present study. In a semi-quantitative study, Peters et al. (2018) detected PVC, PET (44.1%) and nylon (9.3%) in the GIT of six fish species from the Texas Gulf Coast, USA. Ribeiro et al. (2020) quantified PE (70.24–2352.28 µg/g), PVC (9.72 µg/g), PP (1.59–59.89 µg/g) and PMMA (13.96 and 26.76 µg/g) in sardine (*Sardinops neopilchardus*) sampled from an Australian seafood market. In the Monterey Bay of USA, PE, PP and PVC were quantified in California halibut (*Paralichthys californicus*), Chub mackerel (*Scomber japonicus*), Northern lampfish (*Stenobrachius leucopsarus*) and King salmon (*Oncorhynchus tshawytscha*) with combined concentrations of 0.011–87.76, 0.009–11.12, 0.014–1009.11 and 54.21–1,096.43 µg/g, respectively (Hermabessiere et al., 2023). Another study investigating MPs in wild animals of the Sotra Archipelago, Norway, quantified PVC (1.2–3.4 µg/g) and polystyrene (1.0 µg/g) in the Atlantic cod (*Gadus morhua*) and ice flounder (*Limanda limanda*) (Haave et al., 2021).

More recently, archived lanternfishes of 1999–2017 museum collections from the Brazilian portion of South Atlantic Central waters were reported to contain PE (445.47 µg/g), PP (53.7 µg/g), polystyrene (32.4 µg/g) and PET (1.56 µg/g) in their GITs (Ferreira et al., 2025). These concentrations, excluding that of PE in *Sardinops neopilchardus*, archived lanternfishes and the combined polymer concentrations for fish sampled from the Monterey Bay, are comparable to those quantified in the present study. The PE in the cited studies was quantified using C10–C17 alk-1-enes, which might be affected by residual matrix and could partly account for the high concentrations reported.

Considered together, it should be mentioned that some severe analytical challenges still exist when MPs in environmental samples are quantified by Pyr-GC-MS. Firstly, the building of calibration curves with diagnostic pyrolysates of microplastic standards prepared with neat polymers assumes a similar pyrolytic behaviour in both microplastic standards and environmental samples. This is not often the case when dealing with multiple polymers as in the present study. Ultraviolet light aging, for example, has been proven to reduce the yields of PP, PVC, PMMA and PE markers (Ainali et al., 2025; Toapanta et al., 2021), implying that environmental samples with photoaged MPs of these polymers may not be unequivocally detected, or in the best case, may be underestimated. Secondly, differences in molecular weights and stereoregularity of polymers have also been discussed as important determinants of pyrolysate yields (Chen et al., 2025; Iedema et al., 2021), and environmental MPs might present diverse patterns of molecular weight distributions and tacticity, which can differ from the neat polymers used to build calibration curves (see the earlier discussion on methyl substituents and stereoregularity of PP, and their influence on the resultant propylene oligomers). Lastly, the environmental concentrations of some polymers (for example, acrylonitrile butadiene styrene, polycarbonate and polystyrene) are generally much lower, which may explain their non-detection in environmental samples.

### Methodological considerations for PP quantification and risk interpretation

The present study identified an important analytical limitation in the quantification of PP MPs using Pyr-GC-MS. To our knowledge, this is the first mention of an extensive propylene oligomer interference that may lead to false negatives as well as incorrect quantification of PP when a single oligomer is used for the detection and quantification of PP. Thus, the measured PP concentrations in the current study are conservative and should not be interpreted as definitive evidence of low PP microplastic exposure in the GIT of the endemic fish species.

This analytical limitation has implications for polymer-specific risk interpretation, since the assessment of an environmental risk is often based on the abundance, composition and concentration of MPs. Risk interpretation relies on polymer-specific risk rankings based on the quantified concentrations; therefore, an underestimation of PP concentrations may also lead to an underestimated relative risk contribution compared to the other polymers with stable diagnostic pyrolysates.

Future studies should therefore consider performing (i) matrix-matched calibration, (ii) quantification based on the summed peak areas of the propylene oligomers or (iii) standard addition to improve confidence in PP quantification from complex biological and related microplastic-contaminated matrices.

## Conclusion

Starting from the hypothesis that spatiotemporal variations, trophic level, habitat, feeding habit and guild may influence MPs ingestion, the present study assessed MPs contamination of three endemic fish species from L. Victoria. Irrespective of the trophic position, location and season, MPs were only detected in the GIT of gastric fish species. Blue- and brown-coloured 0.3–1.9-mm fragments and filaments were the most dominant, which on subjection to µ-FTIR analysis were found to compose of nylons, PE and PP. Using the refined Pyr-GC-MS method, N6, N66, PP, SBR and PET were quantified for the first time in the GIT of *P. aethiopicus* and *S. victoriae* from L. Victoria.

Polypropylene in the fish samples produced several higher propylene oligomers during pyrolysis, which reduced the relative yield of its target quantification marker and led to false negatives. This result revealed the limitation of calibration approaches based on neat polymer standards, as pyrolytic behaviour may differ in environmental samples containing multiple and weathered polymers in complex matrices. At the same time, the present study highlighted the strength of Pyr-GC-MS, as qualitative inspection of pyrograms allowed polymer identification beyond single quantification markers. In several samples, PP was identified by the presence of characteristic propylene oligomers, even when the target quantification marker (2,4-dimethyl-1-heptene) was absent or below LOD.

## Supplementary Information

Below is the link to the electronic supplementary material.

ESM 1 PDF (702 KB)

## Data Availability

Data supporting the conclusions of this study are available within this article and its supplementary information.
