# Supplementary material for: Refined analysis of microplastics in endemic fish species from Lake Victoria using µ-FTIR and pyrolysis GC-MS
Source: Environ Monit Assess. 2026 Aug 14;198(9):944. doi: 10.1007/s10661-026-15765-5 (PMC13473337; doi:10.1007/s10661-026-15765-5)
Supplement: Supplementary file 1 [file 10661_2026_15765_MOESM1_ESM.pdf]

SUPPORTING INFORMATION

Refined analysis of microplastics in endemic fish species from Lake Victoria using µ-FTIR and Pyrolysis GC-MS

Timothy Omara [a,b], Barbora Benetková [a], Ivan Sumerskii [c], Patrick Ssebugere[b], Christine Kyarimpa[d], Solomon Omwoma Lugasi [e], Thomas Rosenau [a,f], Christine Betty Nagawa [g], Stefan Böhmdorfer [a*]

[a] Institute of Chemistry of Renewable Resources, Department of Natural Sciences and Sustainable Resources, BOKU University, Konrad-Lorenz-Straße 24, 3430 Tulln, Austria

[b] Department of Chemistry, College of Natural Sciences, Makerere University, P.O. Box 7062, Kampala, Uganda

[c] Core Facility Analysis of Lignocellulosics (ALICE), BOKU University, Konrad-Lorenz-Straße 24, 3430 Tulln, Austria

[d] Department of Chemistry, Faculty of Science, Kyambogo University, P.O. Box 1, Kampala, Uganda

[e] Department of Physical Sciences, Jaramogi Oginga Odinga University of Science and Technology, P.O. Box 210-40601, Bondo, Kenya

[f] Johan Gadolin Process Chemistry Centre, Åbo Akademi University, Porthansgatan 3, 20500 Åbo/Turku, Finland

[g] Department of Forestry, Biodiversity and Nature Conservation, College of Agricultural and Environmental Sciences, Makerere University, P.O. Box 7062, Kampala, Uganda

*Corresponding author: stefan.boehmdorfer@boku.ac.at

Table of contents

Table S1. Description of fish species sampled from selected fish landing beaches of Lake Victoria 3

Table S2. Count of microplastics in the gastrointestinal tracts of endemic fish species from Lake Victoria 4

Table S3. Morphometric data of fish samples from Lake Victoria in comparison with previous studies 6

Table S4. Count of microplastic morphologies in the gastrointestinal tracts of *P. aethiopicus* and *S. victoriae* 8

Fig. S1. Size categories of microplastics identified in *Protopterus aethiopicus* by count: [WS] = Wet season, and [DS] = Dry season. 9

Fig. S2. Size categories of microplastics in *Synodontis victoriae* by count: [WS] = Wet season, and [DS] = Dry season. 9

Table S5. Polymers identified by µ-FTIR in the gastrointestinal tracts of endemic fish species from Lake Victoria 10

Fig. S3. µ-FTIR spectrum of a polypropylene particle identified in the gastrointestinal tract of *P. aethiopicus*. The wavenumbers of prominent bands are indicated by arrows. 11

Fig. S4. Pyrogram of the MPs-SiO_2 standard (0.8 mg) prior to post column backflush installation. The last peak of interest is highlighted. SSS = styrene trimer (2,4,6-Triphenyl-1-hexene). Abbreviations of the other pyrolysates and polymers are defined in Table 2. 12

Fig. S5. Pyrogram of the MPs-$CaCO_3$ standard (0.8 mg) prior to post column backflush installation. The pyrolysate MDA (4,4'-Methylenedianiline) is for polyurethane. The styrene trimer (SSS; 2,4,6-Triphenyl-1-hexene) was the last peak of interest. Abbreviations of the other pyrolysates and polymers are defined in Table 2. 13

Fig. S6. Car tires sited near Ripon's Pier, Jinja during trawling of surface water from Lake Victoria in 2023. Encircled in the right foreground is a discarded poly(ethylene terephthalate) water bottle. 13

Table S6. Propylene oligomers, rearrangement products and other pyrolysates identified in pyrograms of fish gastrointestinal tracts 14

Fig. S7. Pyrogram of a 3D printing filament showing clusters of propylene oligomers detected. Propylene (monomer) was also detected, which was not observed for the fish gastrointestinal tracts. 17

References 18

Table S1. Description of fish species sampled from selected fish landing beaches of Lake Victoria

Common name	Binomial name [a]	Feeding habit	Habitat	Trophic level (Natugonza et al., 2016)
Marbled, Ethiopian or leopard lungfish	*Protopterus aethiopicus aethiopicus* Heckel, 1851 [b]	Carnivore	Demersal	3.242
Lake Victoria squeaker	*Synodontis victoriae* Boulenger, 1906	Carnivore	Benthopelagic	3.061
Silver cyprinid/Lake Victoria sardine	*Rastrineobola argentea* (Pellegrin, 1904) [c]	Planktivore	Pelagic	2.052

[a] *All the species studied are of ''Least Concern'' according to the latest assessment by the International Union for Conservation of Nature (IUCN). The species are trophically linked: R. argentea is preyed on by both S. victoriae and P. aethiopicus, while S. victoriae is also preyed upon by P. aethiopicus.*

[b] *This is the nominate subspecies of the Ethiopian lungfish in Lake Victoria. Its gills were not studied because it is an obligate air breather; the internal gills are reduced (fewer filaments and lamellae) and less functional for respiration in adults.*

[c] *An opportunistic omnivore. Its gills are very small and could not be isolated for microplastic extraction.*

Table S2. Count of microplastics in the gastrointestinal tracts of endemic fish species from Lake Victoria

Species	Site	Sample code [a]	Sex	Particle counts	Average/GIT[b]	Median/GIT	Number of particles/g GIT	Average/g GIT [b]	Median (IQR) g/GIT[b]	Pyr-GC-MS code [c]
Protopterus aethiopicus	Ripon Falls	PA-1 RIFA GIT	F	11			0.19			Ripon Falls 01
		PA-2 RIFA GIT	F	12	16.0±7.8	12	0.20	0.35±0.27	0.20 (0.19–0.66)	–
		PA-3 RIFA GIT	F	25			0.66			Ripon Falls 02
		PA-4 RIFA GIT	M	11			0.38			Ripon Falls 03
		PA-5 RIFA GIT	M	3	6.0±4.4	4	0.11	0.20±0.16	0.11 (0.11–0.38)	–
		PA-6 RIFA GIT	M	4			0.11			Ripon Falls 04
	Katosi	PA-1 KAT GIT	M	8			0.25			Katosi 01
		PA-2 KAT GIT	F	6	6.3±1.5	6	0.25	0.24±0.01	0.25 (0.23–0.25)	–
		PA-3 KAT GIT	F	5			0.23			Katosi 02
		PA-4 KAT GIT	M	19			0.56			Katosi 03
		PA-5 KAT GIT	M	4	9.3±8.4	5	0.16	0.30±0.22	0.16 (0.19–0.56)	–
		PA-6 KAT GIT	M	5			0.19			Katosi 04
	Port Bell	PA-1 PB GIT	F	6			0.12			Port Bell 01
		PA-2 PB GIT	F	3	5.0±1.7*	6	0.06	0.11±0.04	0.12 (0.06–0.14)*	Port Bell 02
		PA-3 PB GIT	F	6			0.14			–
		PA-4 PB GIT	M	3			0.07			–
		PA-5 PB GIT	F	9	5.7±3.1	5	0.28	0.16±0.11	0.13 (0.07–0.28)	Port Bell 03
		PA-6 PB GIT	M	5			0.13			Port Bell 04
Synodontis victoriae	Ripon Falls	SV-1 RIFA GIT	M	18			3.75			Ripon Falls 01
		SV-2 RIFA GIT	M	19	14.7±6.7	18	5.14	3.48±1.80	3.75 (1.56–5.14)	–
		SV-3 RIFA GIT	M	7			1.56			Ripon Falls 02
		SV-4 RIFA GIT	M	5			1.06			Ripon Falls 03
		SV-5 RIFA GIT	M	3	6.0±3.6	5	0.71	1.27±0.69	1.06 (0.71–2.04)	–
		SV-6 RIFA GIT	F	10			2.04			Ripon Falls 04
	Katosi	SV-1 KAT GIT	F	4			8.00			–
		SV-2 KAT GIT	F	18	11.0±7.0	11	9.00	8.49±0.50	8.43 (8.00–9.00)	Katosi 01
		SV-3 KAT GIT	F	11			8.46			Katosi 02
		SV-4 KAT GIT	F	10			5.88			Katosi 03
		SV-5 KAT GIT	M	9	10.7±2.1*	10	10.00	8.63±2.38	10.00 (5.88–10.0)*	Katosi 04
		SV-6 KAT GIT	M	13			10.00			–
	Port Bell	SV-1 PB GIT	F	5			1.00			Port Bell 01
		SV-2 PB GIT	F	4	4.7±0.6	5	0.89	1.04±0.17	1.00 (0.89–1.22)	Port Bell 02
		SV-3 PB GIT	M	5			1.22			–
		SV-4 PB GIT	F	10			2.86			–
		SV-5 PB GIT	M	6	9.3±3.1	10	1.43	2.97±1.60	2.86 (1.43–4.62)	Port Bell 03
		SV-6 PB GIT	M	12			4.62			Port Bell 04

[a] *Sex: M = Male, F = Female, GIT = Gastrointestinal tract.*

[b] *Average ± standard deviation of replicates. Sample codes 1-3, and 4-6 refer to dry and wet seasons, respectively. Medians marked with an asterisk (*) in a given column are statistically different (H = 31.7, adjusted P = 0.01). IQR = Interquartile range.*

[c] *Codes refer to samples with polymer concentrations obtained by Pyr-GC-MS (**Table 3**);* – means *μ-FTIR sample (**Table S5**).*

Text S1. Morphometric data of fish samples

The average total length of *P. aethiopicus* ranged from 54.3±2.3 cm at Katosi during the wet season to 71.5±3.5 cm in dry season fish from Ripon Falls, while the standard lengths were from 35.5±3.8 cm for dry season samples at Katosi to 48.0±3.5 cm for dry season samples from Ripon Falls (**Table S3**). The highest average wet weight was 1107.0±327.2 g for the longest samples obtained from Ripon Falls during the dry season. Statistically, there were significant differences in the total and standard lengths (P = 0.002 in each case), as well as wet weights of *P. aethiopicus* across the FLBs (P = 0.0006). Badamasi et al. (2020) had average total lengths and wet weights of 56.5 and 55.7 cm, and 845.3 and 1,102.3 g for male and female *P. aethiopicus* sampled from Jinja, Port Bell, Ggaba, Bukakata, Kasensero and Bale FLBs of L. Victoria. In Mwanza (Tanzania), the average lengths of *P. aethiopicus* at maturity was 96 cm (Mosille & Mmnoya, 1988), 65 to 76 cm for females and 82 cm for males (Greenwood, 1958; Okedi, 1971). In Lake Baringo of Kenya where *P. aethiopicus* was introduced in 1975, the established fish has been reported to grow up to lengths of 17.5 to 42.5 cm (Goudswaard et al., 2002), 58 to 102 cm (Mlewa et al., 2005) and 43.0 to 145.0 cm, with weights of up to 17500 g (Mlewa & Green, 2004), which are relatively higher than reported in the present study.

The silver cyprinid (*R. argentea*) had total and standard body lengths ranging from 4.8±0.6 cm and 3.3±0.4 cm at Katosi during the wet season to 5.8±0.3 cm and 4.2±0.4 cm at Ripon Falls during the dry season, respectively. On the other hand, the wet weights varied from 0.25±0.04 g for fish from Ripon Falls sampled during the wet season to 0.60±0.11 g for those from Port Bell obtained during the dry season. For this species, only wet weights were significantly influenced by both season and sampling location as per Two-Way ANOVA (P = 0.0002). The *R. argentea* sampled in this study had longer total and standard lengths when compared to previous studies in L. Victoria (Aisu, 2022; Clarke et al., 2022; Poste, 2010; Yongo et al., 2016; Yongo et al., 2019) and Lake Bulera, Rwanda (Isumbisho et al., 2011). In Lake Ruhondo (Rwanda) which it invaded, *R. argentea* total lengths varied from 3.4 to 9.9 cm (Isumbisho et al., 2011) and this is higher than observed in the present study. It was cited that the length of *R. argentea* in Northern L. Victoria at first maturity is 4.7 cm (Wandera, 1990), and mature adults hardly reach lengths more than 9 cm (Eccles, 1992).

The total and standard lengths of *S. victoriae*, varied from 19.2±1.3 to 27.6±3.0 cm, and 15.6±0.7 to 22.2±2.5 cm for samples from Port Bell taken during the two seasons (P = 0.001 in each case). Similar to *P. aethiopicus*, the longest *S. victoriae* samples also had the highest mean wet weight (197.0±55.8 g). The mean weights were statistically different among samples (P = 0.01). *Synodontis victoriae* generally grows to a maximum of 35 cm (Froese & Pauly, 2016), and according to Bishai and Abu Gideiri (1965), female fish from the *Synodontis* genus are slightly larger than males of the same age. Taken together, morphometric and meristic characteristics of fish respond to environmental factors, which may vary among different and the same species. Our results agreed with Mwanja et al. (2011) who postulated that morphological changes and divergence within a species are expected in L. Victoria for fish that get exposed to new developmental and evolutionary pressures. This is specifically true for *R. argentea* that has shown typical r-selected life strategy in response to increased predation and fishing through reducing its size at maturity (Manyala & Ojuok, 2007). Such reduced growths as a predator defense mechanism were also reported in the cyprinid carp (*Carassius carassius*)(Olsén & Bonow, 2023).

Table S3. Morphometric data of fish samples from Lake Victoria in comparison with previous studies

Species	Sampling location	Total length (cm)	Standard length (cm)	Wet weight (g)	References
P. aethiopicus	Ripon Falls (Dry season)	71.5±3.5 (68.9–75.5) [a]	48.0±3.5 (44.0–50.6) [a]	1107.0±327.2 (797.0–1449.0) [a]	Current study
	Ripon Falls (Wet season)	59.6±6.1 (54.8–66.4) [b,c]	38.4±4.1 (35.4–43.1) [b,c]	769.3±31.3 (736.0–798.0) [a,b,c]	
	Katosi (Dry season)	57.2±4.5 (3.1–4.1) [b,c]	35.5±3.8 (32.5–38.0) [c]	527.3±20.3 (504.0–541.0) [c]	
	Katosi (Wet season)	54.3±2.3 (52.3–56.9) [c]	37.7±1.9 (36.2–39.8) [b,c]	461.3±30.8 (437.0–496.0) [c]	
	Port Bell (Dry season)	67.7±1.6 (66.5–69.5) [a,b]	42.2±0.8 (41.5–43.0) [a,b,c]	977.3±66.4 (937.0–1054.0) [a,b]	
	Port Bell (Wet season)	63.1±4.0 (60.1–67.6) [a,b,c]	43.7±2.8(40.9–46.5) [a,b]	719.0±35.2 (679.0–745.0) [b,c]	
	White Nile, Ethiopia	29.8–52.4	–	–	Orlov et al. (2015)
	Thruston Bay, L. Victoria	52.1	–	1770	Muyodi et al. (2011)
	Lake Edward, Uganda	–	51.0–83.0	–	Poste (2010)
	Lake George, Uganda	–	47.0–82.5	–	
	Lake Mburo, Uganda	–	53.0–102.0	–	
	Murchison Bay, L. Victoria	–	37.7–79.0	–	
	Napoleon Gulf, L. Victoria	–	50–100	–	
	Lake Nabugabo, Uganda	–	–	44–222	Seifert and Chapman (2007)
	Lake Baringo, Kenya	61.0–81.0	–	750–2000	Mlewa et al. (2007)
	Thruston Bay and Napoleon Gulf, L. Victoria	72.0	–	450	Campbell et al. (2004)
	Open lake, L. Victoria	43.0–130.0	–	–	EAHC (1959)
R. argentea	Ripon Falls (Dry season)	5.8±0.3 (5.5–6.1) [a]	4.2±0.4 (3.8–4.6) [a]	0.28±0.03 (0.25–0.31) [c]	Current study
	Ripon Falls (Wet season)	5.7±0.5 (5.2–6.1) [a]	4.1±0.5 (3.6–4.5) [a]	0.25±0.04 (0.21–0.29) [c]	
	Katosi (Dry season)	5.0±0.5 (4.6–5.5) [a]	3.5±0.5 (3.1–4.0) [a]	0.41±0.04 (0.37–0.44) [a,b]	
	Katosi (Wet season)	4.8±0.6 (4.2–5.3) [a]	3.3±0.4 (2.9–3.7) [a]	0.38±0.04 (0.35–0.42) [b,c]	
	Port Bell (Dry season)	4.9±0.9 (4.2–5.9) [a]	3.8±0.3 (3.6–4.1) [a]	0.60±0.11 (0.49–0.70) [a]	
	Port Bell (Wet season)	5.6±0.6 (5.1–6.3) [a]	3.9±0.5 (3.5–4.4) [a]	0.47±0.08 (0.39–0.55) [a,b]	
	Ggaba and Katosi, L. Victoria	–	3.9 and 3.7	–	Aisu (2022)
	Kikondo, L. Victoria	4.0±0.1 (3.3–5.0)	3.4±0.1 (2.8–4.3)	0.5±0.0 (0.2–0.9)	Clark et al. (2022)
	Lambu, L. Victoria	4.2±0.1 (3.5–5.1)	3.5±0.1 (2.9–4.2)	0.6±0.0 (0.3–1.1)	
	Kiyindi, L. Victoria	4.7±0.1 (4.4–5.2)	4.0±0.1 (3.6–4.4)	0.8±0.0 (0.6–1.1)	
	Lake Nabugabo, Uganda	4.7±0.1 (4.4–5.1)	3.9±0.1 (3.6–4.3)	0.7±0.0 (0.5–0.8)	
	Nyanza Gulf, L. Victoria	–	1.7–5.5	0.08–2.44	Yongo et al. (2019)
	Nyanza Gulf, L. Victoria	–	3.5–4.2	–	Yongo et al. (2016)
	Murchison Bay, L. Victoria	–	3.5	–	Poste (2010)

Species	Sampling location	Total length (cm)	Standard length (cm)	Wet weight (g)	References
	Napoleon Gulf, L. Victoria	–	3.5–4.0	–	
	Lake Ruhondo, Rwanda	7.26±1.5 (3.4–9.9)	–	–	Isumbisho et al. (2011)
	Lake Bulera, Rwanda	4.18±0.6 (2.5–6.5)	–	–	
	Pilkington Bay, L. Victoria	–	4.0–4.4	–	Wandera et al. (1990)
	Ripon Falls (Dry season)	19.6±0.4 (19.2–19.9) [b]	15.6±0.9 (15.0–16.6) [b]	159.0±1.73 (157.2–160.0) [a,b]	Current study
	Ripon Falls (Wet season)	19.5±1.4 (18.4–21.0) [b]	15.9±0.6 (15.3–16.5) [b]	161.0±8.0 (153.0–169.0) [a,b]	
	Katosi (Dry season)	22.0±2.9 (19.5–25.2) [b]	17.9±2.6 (15.0–20.0) [b]	98.3±26.6 (73.0–126.0) [b,c]	
	Katosi (Wet season)	20.8±0.3 (20.5–21.1) [b]	15.7±0.3 (15.4–16.0) [b]	86.6±10.7 (85.0–98.0) [c]	
S. victoriae	Port Bell (Dry season)	27.6±3.0 (25.7–31.0) [a]	22.2±2.5 (20.5–25.0) [a]	197.0±55.8 (154.0–260.0) [a]	
	Port Bell (Wet season)	19.2±1.3 (17.7–20.1) [b]	15.6±0.7 (14.9–16.3) [b]	166.0±6.6 (159.0–172.0) [a,b]	
	Kisumu Bay, L. Victoria	–	16.5–17.4	46.8–51.3	Gathu et al. (2024)
	Nyanza Gulf, L. Victoria	–	–	13.0–99.0	Yongo and Wairimu (2018)
	Murchison Bay, L. Victoria	–	17.1–24.5	–	Poste (2010)
	Napoleon Gulf, L. Victoria	–	17.1–23.5	–	

Note: *Results are means ± standard deviations of triplicates. Values in parentheses are ranges. – means not reported. Mean values of morphometric data for a fish species that do not share a superscript letter are statistically different (P<0.05) according to Two-Way ANOVA.*

Table S4. Count of microplastic morphologies in the gastrointestinal tracts of *P. aethiopicus* and *S. victoriae*

Species	Site	Field sample code	Fragments	Fibres	Filaments	Films	Pellets	Foam
Protopterus aethiopicus	Ripon Falls	PA-1 RIFA GIT	8	1	2	0	0	0
		PA-2 RIFA GIT	8	1	2	0	1	0
		PA-3 RIFA GIT	23	1	1	0	0	0
		PA-4 RIFA GIT	7	2	2	0	0	0
		PA-5 RIFA GIT	3	0	0	0	0	0
		PA-6 RIFA GIT	2	1	1	0	0	0
	Katosi	PA-1 KAT GIT	7	0	1	0	0	0
		PA-2 KAT GIT	4	0	2	0	0	0
		PA-3 KAT GIT	2	1	1	0	0	1
		PA-4 KAT GIT	14	3	2	0	0	0
		PA-5 KAT GIT	3	0	0	1	0	0
		PA-6 KAT GIT	4	1	0	0	0	0
	Port Bell	PA-1 PB GIT	4	0	2	0	0	0
		PA-2 PB GIT	2	0	1	0	0	0
		PA-3 PB GIT	2	1	3	0	0	0
		PA-4 PB GIT	1	1	1	0	0	0
		PA-5 PB GIT	4	1	4	0	0	0
		PA-6 PB GIT	4	0	1	0	0	0
Synodontis victoriae	Ripon Falls	SV-1 RIFA GIT	10	5	3	0	0	0
		SV-2 RIFA GIT	14	4	1	0	0	0
		SV-3 RIFA GIT	6	0	1	0	0	0
		SV-4 RIFA GIT	4	0	0	1	0	0
		SV-5 RIFA GIT	2	0	0	1	0	0
		SV-6 RIFA GIT	8	0	2	0	0	0
	Katosi	SV-1 KAT GIT	3	0	1	0	0	0
		SV-2 KAT GIT	12	0	6	0	0	0
		SV-3 KAT GIT	6	3	2	0	0	0
		SV-4 KAT GIT	6	2	2	0	0	0
		SV-5 KAT GIT	4	2	0	3	0	0
		SV-6 KAT GIT	11	1	1	0	0	0
	Port Bell	SV-1 PB GIT	3	0	2	0	0	0
		SV-2 PB GIT	2	1	1	0	0	0
		SV-3 PB GIT	5	0	0	0	0	0
		SV-4 PB GIT	9	1	0	0	0	0
		SV-5 PB GIT	6	0	0	0	0	0
		SV-6 PB GIT	7	0	5	0	0	0

Note: *Sample codes 1-3, and 4-6 refer to dry and wet seasons samples, respectively.*

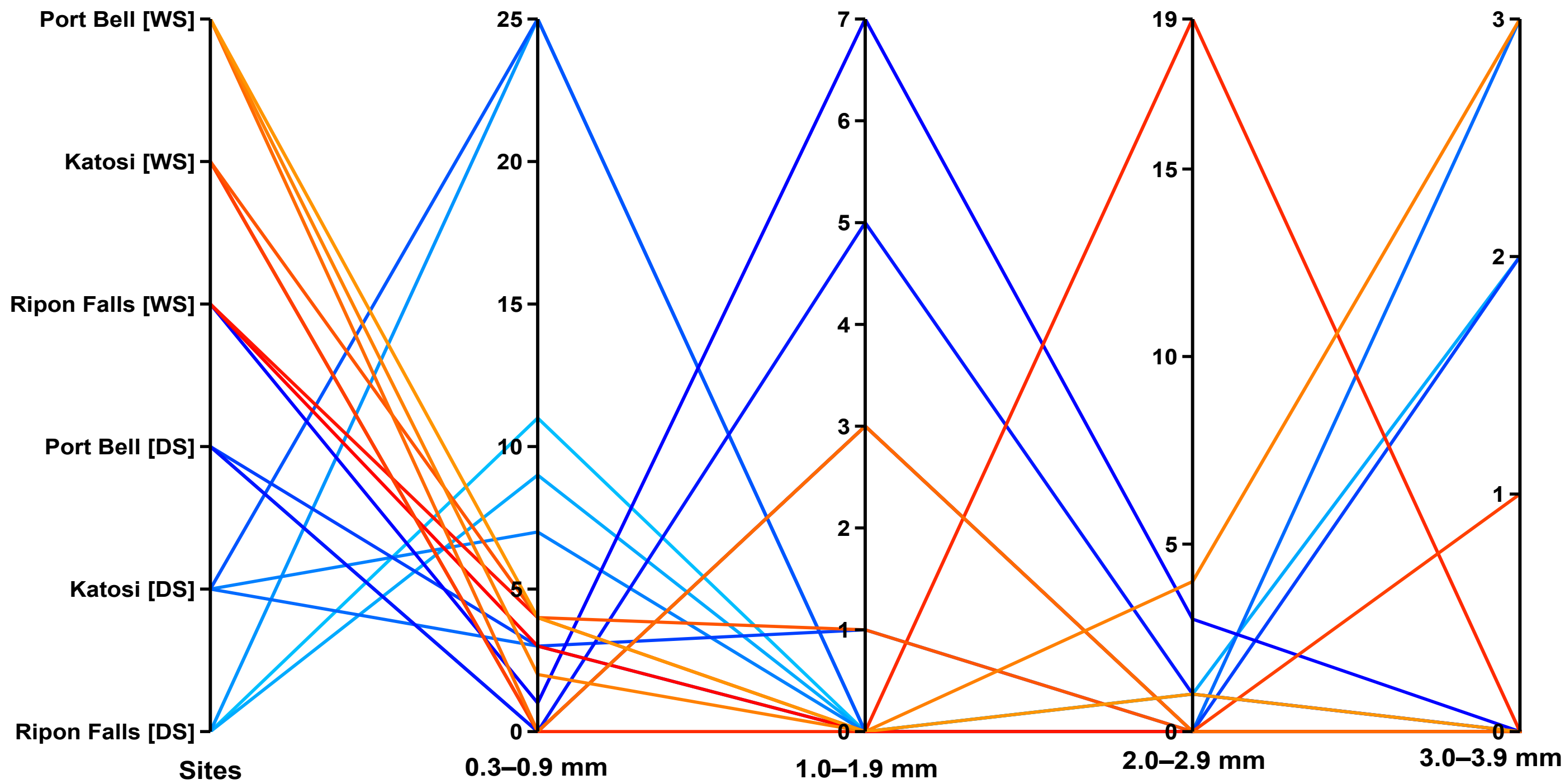

Fig. S1. Size categories of microplastics identified in *Protopterus aethiopicus* by count: [WS] = Wet season, and [DS] = Dry season.

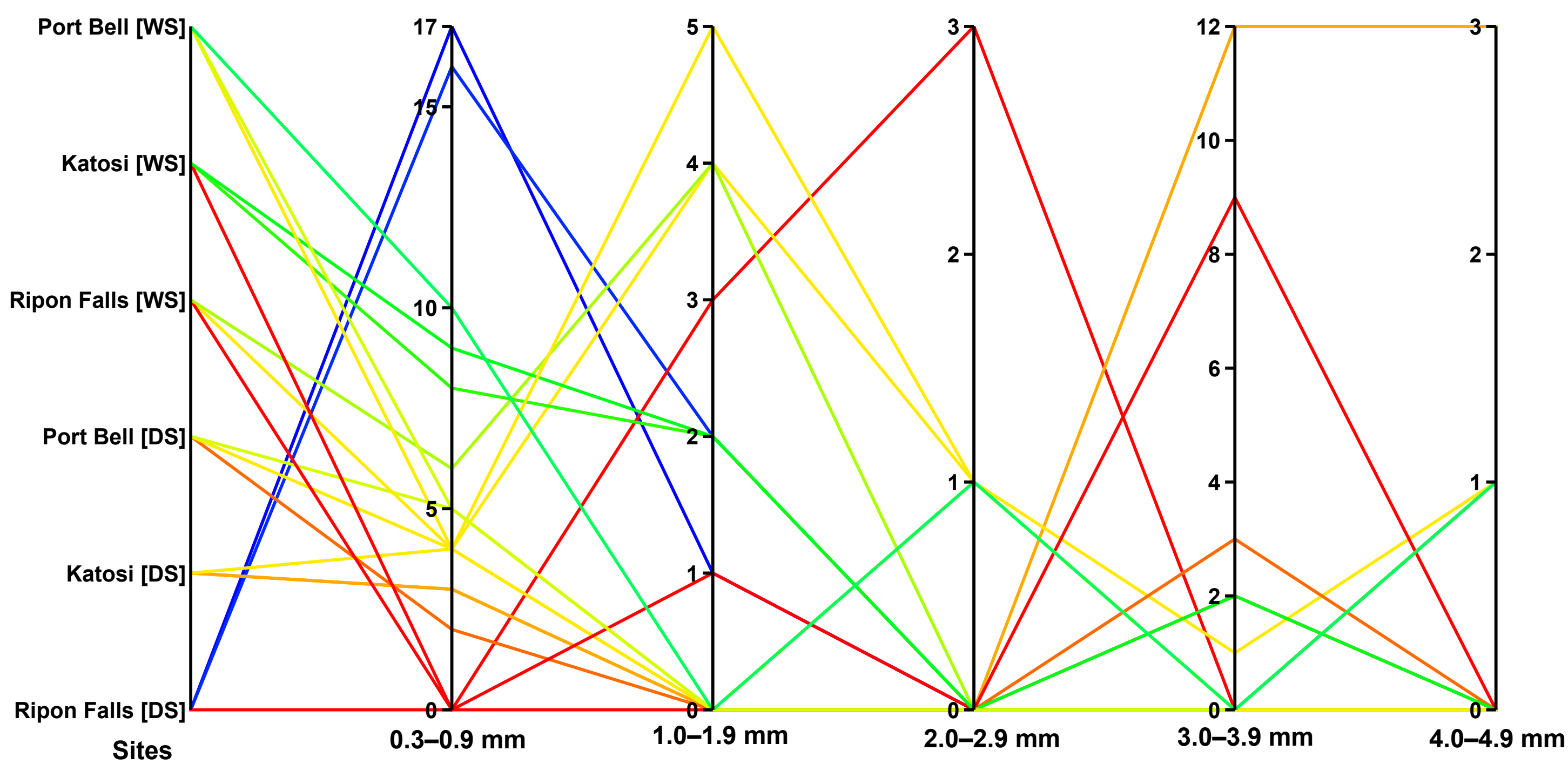

Fig. S2. Size categories of microplastics in *Synodontis victoriae* by count: [WS] = Wet season, and [DS] = Dry season

Table S5. Polymers identified by µ-FTIR in the gastrointestinal tracts of endemic fish species from Lake Victoria

Sampling site (season)	Species	Sample code	FTIR No.	Spectrum No.	Polymers identified	Scores (%)
Ripon Falls (Dry season)	*Protopterus aethiopicus*	PA-2 RIFA GIT	25	25 FTIR-1	Polypropylene (isotactic)	78.9
				25 FTIR-2	Nylon 6/66	67.8
				25 FTIR-3	Nylon 6/66	65.7
Ripon Falls (Wet season)		PA-5 RIFA GIT	26	26 FTIR-1	Nylon 6/66	65.0
				26 FTIR-2	Nylon 6/66	65.4
				26 FTIR-3	Nylon 6/66	65.6
				26 FTIR-4	Nylon 6/10	64.6
				26 FTIR-5	*Cellulose* [a]	*64.3*
Katosi (Dry season)		PA-2 KAT GIT	27	27 FTIR-1	Polyethylene	66.1
				27 FTIR-2	Polyethylene	66.8
Katosi (Wet season)		PA-5 KAT GIT	28	28 FTIR-1	*Cellulose*	*64.5*
				28 FTIR-2	*Cellulose*	*72.4*
				28 FTIR-3	*Cellulose*	*72.5*
				28 FTIR-4	*Cellulose*	*72.2*
Port Bell (Dry season)		PA-3 PB GIT	29	29 FTIR-1	Nylon (amorphous)	65.7
				29 FTIR-2	Nylon (amorphous)	65.1
				29 FTIR-3	*Cellulose (cotton)*	*77.1*
				29 FTIR-4	Nylon (amorphous)	65.3
Port Bell (Wet season)		PA-4 PB GIT	30	30 FTIR-1	Polypropylene (isotactic)	93.3
				30 FTIR-2	Nylon 6/66	73.5
				30 FTIR-3	Nylon 6/66	73.6
				30 FTIR-4	Nylon 6/10	74.1
Ripon Falls (Dry season)	*Synodontis victoriae*	SV-2 RIFA GIT	31	31 FTIR-1	Nylon 66	57.8[b]
				31 FTIR-2	Hydroxypropyl cellulose	56.2[c]
				31 FTIR-3	Nylon 11 (Polyundecanoamide)	57.6[b]
Ripon Falls (Wet season)		SV-5 RIFA GIT	32	32 FTIR-1	Nylon 11 (Polyundecanoamide)	64.8
				32 FTIR-2	Nylon 11 (Polyundecanoamide)	65.2
				32 FTIR-3	Polypropylene (isotactic)	91.0
Katosi (Dry season)		SV-1 KAT GIT	33	33 FTIR-1	Polyethylene	62.5
				33 FTIR-2	Polyethylene	62.7
Katosi (Wet season)		SV-6 KAT GIT	34	34 FTIR-1	Nylon 11 (Polyundecanoamide)	64.7
				34 FTIR-2	Nylon 11 (Polyundecanoamide)	64.7
				34 FTIR-3	Nylon 11 (Polyundecanoamide)	64.8
				34 FTIR-4	Nylon 11 (Polyundecanoamide)	63.5
Port Bell (Dry season)		SV-3 PB GIT	35	35 FTIR-1	Nylon 11 (Polyundecanoamide)	55.7[b]
				35 FTIR-2	Nylon 66	61.3
				35 FTIR-3	Nylon (amorphous)	63.0
Port Bell (Wet season)		SV-4 PB GIT	36	36 FTIR-1	Nylon (amorphous)	63.2
				36 FTIR-2	Nylon (amorphous)	63.5

[a] *Natural polymer (italicised).*

[b] *Polymer confirmed in KnowItAll software; the indicated score is from LabSolutions IR library matching.*

[c] *Excluded since it had a score less than 60% in all library searches.*

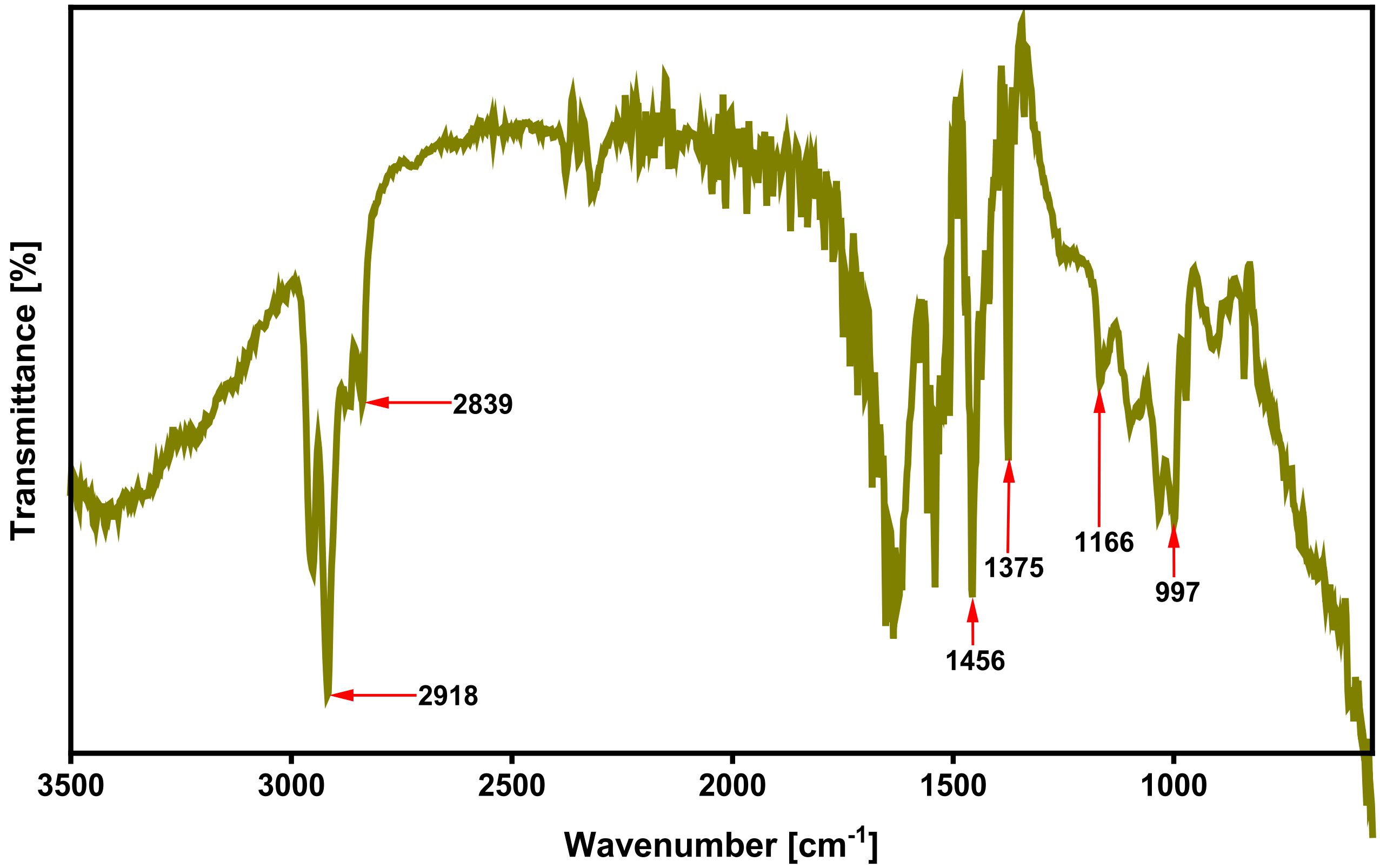

Fig. S3. µ-FTIR spectrum of a polypropylene particle identified in the gastrointestinal tract of *P. aethiopicus*. The wavenumbers of prominent bands are indicated by arrows.

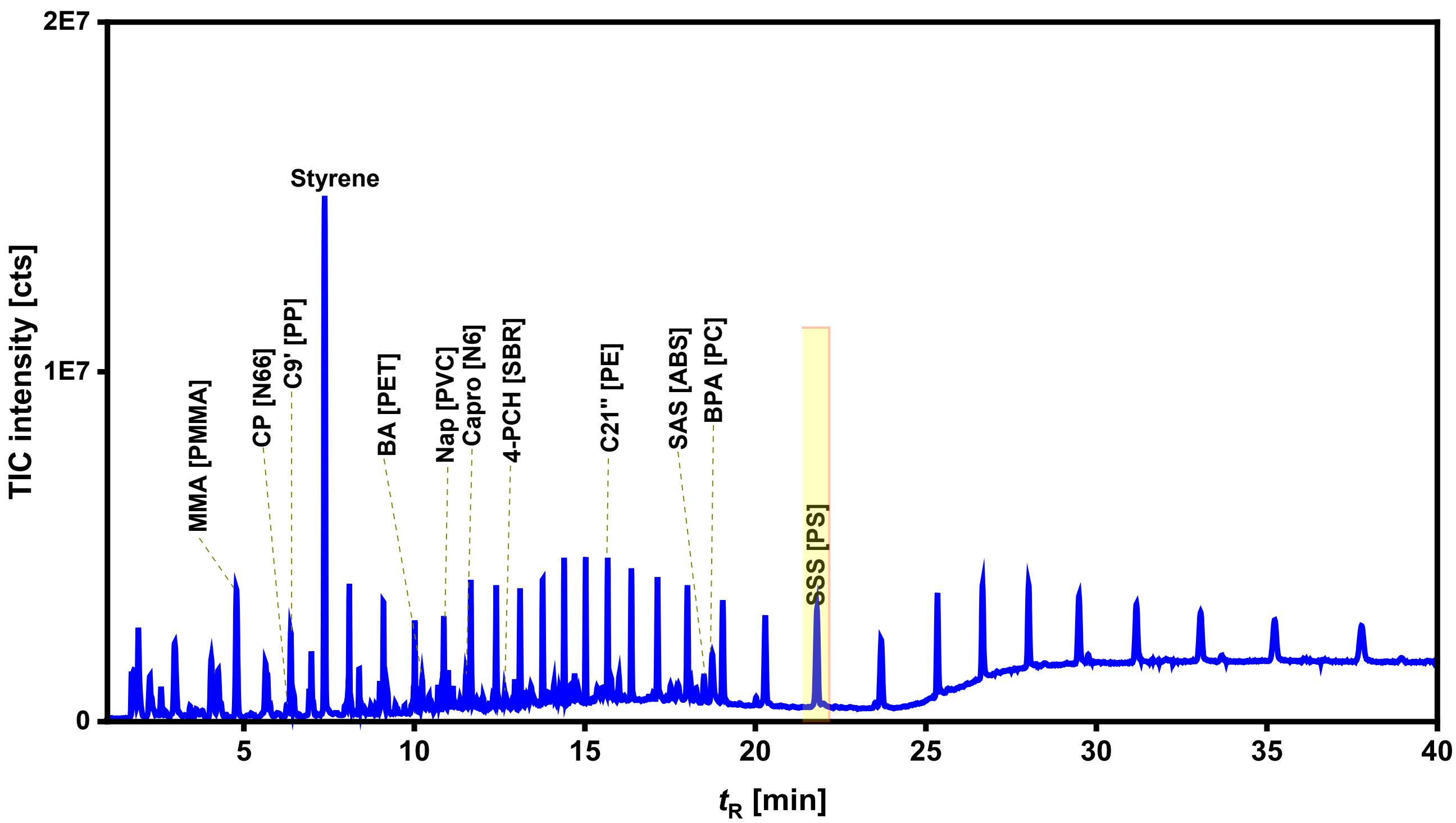

Fig. S4. Pyrogram of the MPs-SiO_2 standard (0.8 mg) prior to post column backflush installation. The last peak of interest is highlighted. SSS = styrene trimer (2,4,6-Triphenyl-1-hexene). Abbreviations of the other pyrolysates and polymers are defined in **Table 2**.

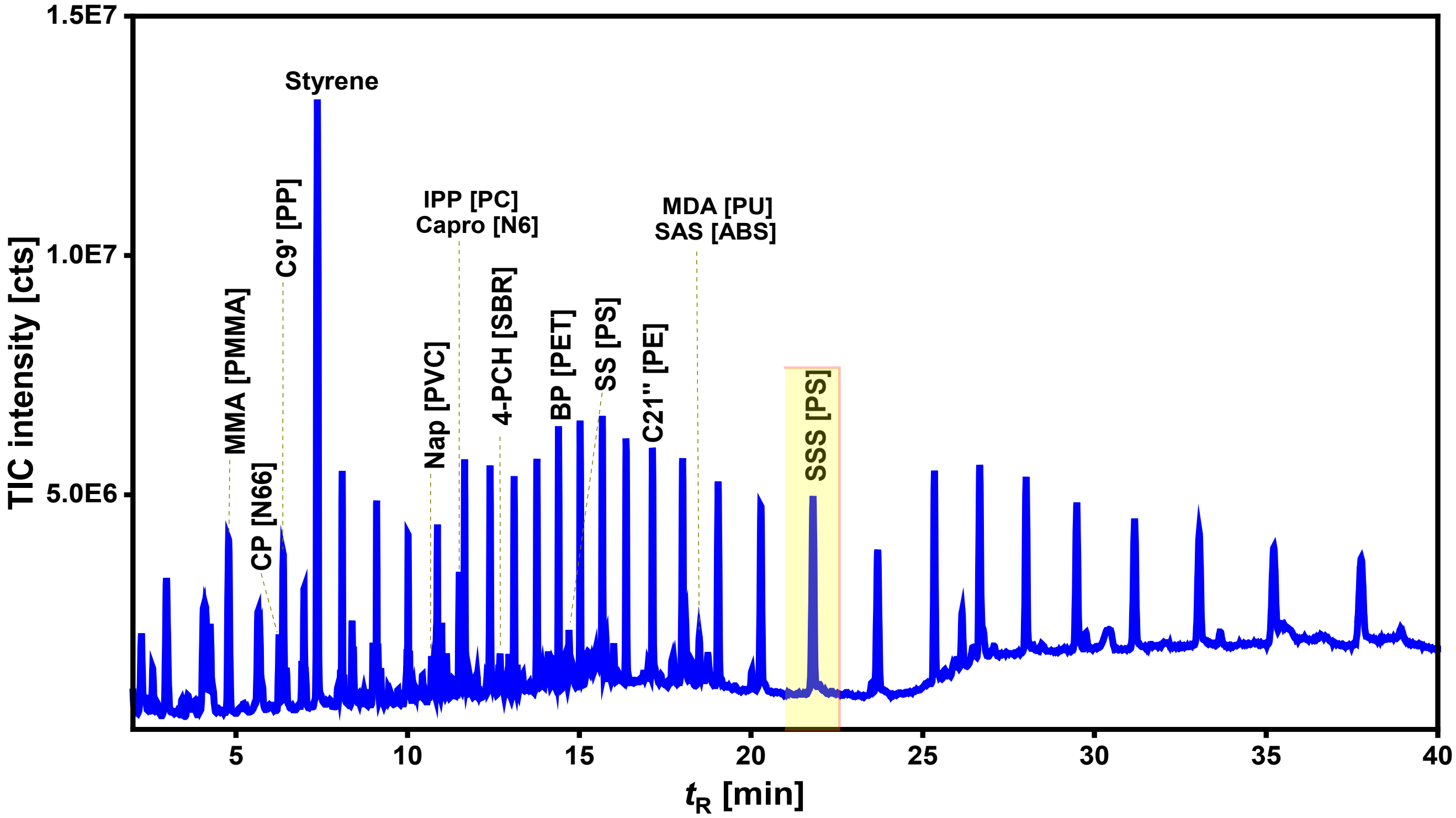

Fig. S5. Pyrogram of the MPs-$CaCO_3$ standard (0.8 mg) prior to post column backflush installation. The pyrolysate MDA (4,4'-methylenedianiline) is for polyurethane. The styrene trimer (SSS; 2,4,6-Triphenyl-1-hexene) was the last peak of interest. Abbreviations of the other pyrolysates and polymers are defined in **Table 2**.

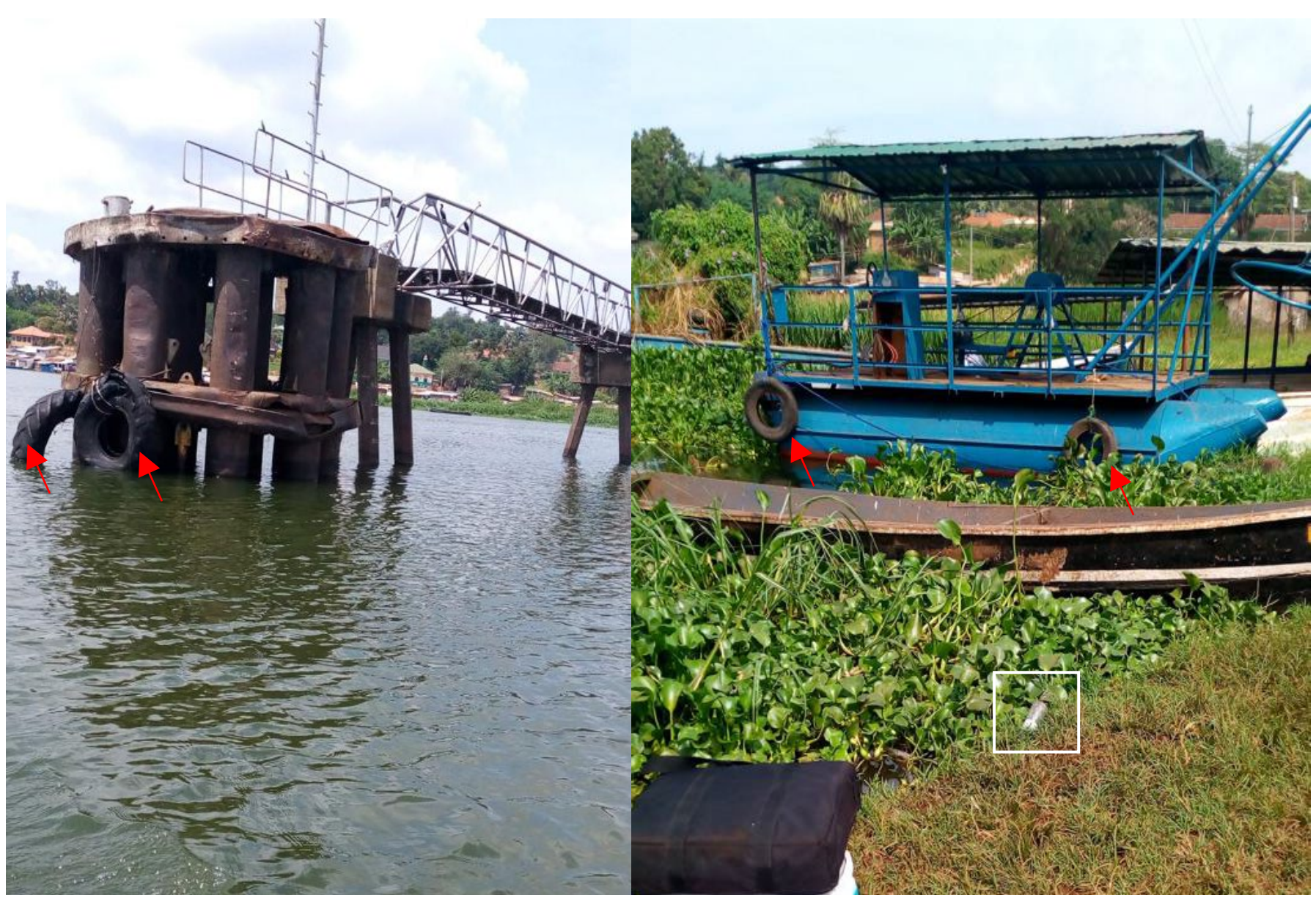

Fig. S6. Car tires sited near Ripon's Pier, Jinja during trawling of surface water from Lake Victoria in 2023. Encircled in the right foreground is a discarded poly(ethylene terephthalate) water bottle.

Table S6. Propylene oligomers, rearrangement products and other pyrolysates identified in pyrograms of fish gastrointestinal tracts

Analytical disc pair code	Field code	Fish landing beach (season)	Pyrolysates
MPs45_PA01	PA-1 RIFA GIT	Ripon Falls 01 (Dry season)	Propylene trimer (3,4,5-Trimethyl-1-hexene), pentamer (2,4,6,8-Tetramethyl-1-undecene), naphthalene, styrene, 3-Methyl-1,6-heptadiene, benzoic acid
MPs45_PA03			Propylene trimers (2,4-Dimethyl-1-heptene and 2,2-Dimethyl-3-heptene), tetramers (2,3,3-Trimethyl-4-nonene and 4,6,8-Trimethyl-1-nonene), 2,2,4-Trimethyl-3-hexene, naphthalene, benzoic acid
MPs45_PA04			Propylene trimer (2,4-Dimethyl-1-heptene), tetramers (4,6,8-Trimethyl-1-nonene and 2,2,3,5,6-Pentamethyl-3-heptene), conjugated diene (3,3,6-Trimethyl-1,4-heptadiene), naphthalene, benzoic acid
MPs46_PA01	PA-3 RIFA GIT	Ripon Falls 02 (Dry season)	Cyclopentanone, benzoic acid
MPs47_PA03	PA-4 RIFA GIT	Ripon Falls 03 (Wet season)	Propylene dimer (2-Methyl-1-pentene), trimer (2,4-Dimethyl-1-heptene), tetramer (4,6,8-Trimethyl-1-nonene), 2,2,4-Trimethyl-3-hexene, cyclopentanone
MPs47_PA04			Propylene trimer (2,4-Dimethyl-1-heptene), tetramer (4,6,8-Trimethyl-1-nonene)
MPs48_PA02	PA-6 RIFA GIT	Ripon Falls 04 (Wet season)	Cyclopentanone, benzoic acid
MPs49_PA02	PA-1 KAT GIT	Katosi 01 (Dry season)	2,2,5,5-Tetramethyl-3-hexene, cyclopentanone, naphthalene, benzoic acid
MPs49_PA03			Propylene trimer (2,4-Dimethyl-1-heptene), 2-Methyl-1-butene, cyclopentanone, naphthalene, benzoic acid
MPs50_PA01 to MPs50_PA03	PA-3 KAT GIT	Katosi 02 (Dry season)	Cyclopentanone, naphthalene
MPs51_PA01 to MPs51_PA04	PA-4 KAT GIT	Katosi 03 (Wet season)	2-Methyl-1-butene, cyclopentanone, naphthalene
MPs52_PA02	PA-6 KAT GIT	Katosi 04 (Wet season)	Cyclopentanone, naphthalene
MPs52_PA03			2-Methyl-1-butene
MPs52_PA04			2-Methyl-1-butene, cyclopentanone, naphthalene
MPs53_PA01	PA-1 PB GIT	Port Bell 01 (Dry season)	Propylene dimer (2-Methyl-1-pentene), trimer (2,4-Dimethyl-1-heptene), cyclopentanone, naphthalene, benzoic acid
MPs53_PA03	PA-1 PB GIT		Propylene dimer (2-Methyl-1-pentene), trimer (2,4-Dimethyl-1-heptene), pentamer (2,4,4,6,6,8,8-Heptamethyl-2-nonene), cyclopentanone, naphthalene, benzoic acid
MPs54_PA01 to MPs54_PA03	PA-2 PB GIT	Port Bell 02 (Dry season)	2-Methyl-2-butene, cyclopentanone, naphthalene
MPs54_PA04			Benzophenone, naphthalene
MPs55_PA01	PA-5 PB GIT	Port Bell 03 (Wet season)	Propylene dimer (3-Methyl-2-pentene), trimer (2,4-Dimethyl-1-heptene), tetramer (4,6,8-Trimethyl-1-nonene), 4-Vinylcyclohexene, 4-Phenylcyclohexene, styrene, α-Methyl styrene, cyclopentanone, naphthalene, benzoic acid

MPs55_PA03			Propylene dimer (2-Methyl-1-pentene), styrene, naphthalene, benzoic acid, 4-Phenylcyclohexene
MPs56_PA04	PA-6 PB GIT	Port Bell 04 (Wet season)	Propylene dimer (4-Methyl-1-pentene), cyclopentanone, styrene, naphthalene, benzoic acid
MPs57_SV02	SV-1 RIFA GIT	Ripon Falls 01 (Dry season)	Propylene tetramer (2,4,4,6-Tetramethyl-2-heptene), cyclopentanone, naphthalene, caprolactam, benzoic acid, 4-Phenylcyclohexene
MPs58_SV01	SV-3 RIFA GIT	Ripon Falls 02 (Dry season)	Propylene dimer (2-Methyl-1-pentene), 2-Methyl-1-butene, 4,4-Dimethyl-1-hexene, cyclopentanone, naphthalene, caprolactam benzoic acid
MPs58_SV03			Propylene trimer (2,6-Dimethyl-1-heptene), 2,4-Dimethyl-1-decene, cyclopentanone, benzoic acid, caprolactam
MPs59_SV02	SV-4 RIFA GIT	Ripon Falls 03 (Wet season)	Propylene dimer (2-Methyl-1-pentene), trimer (2,4-Dimethyl-1-heptene), tetramers (2,4,6-Trimethyl-1-nonene and 4,6,8-Trimethyl-1-nonene), pentamer (2,4,6,8-Tetramethyl-1-undecene), cyclopentanone, naphthalene, benzoic acid, caprolactam
MPs59_SV03			Propylene trimers (2,3-Dimethyl-3-heptene and 2,4-Dimethyl-1-heptene), tetramer (2,4,6-Trimethyl-1-nonene), pentamer (2,4,6,8-Tetramethyl-1-undecene), benzoic acid, caprolactam
MPs60_SV03	SV-6 RIFA GIT	Ripon Falls 04 (Wet season)	Propylene pentamer (2,4,6,8-Tetramethyl-1-undecene), cyclopentanone, naphthalene, benzoic acid, caprolactam
MPs60_SV04			Propylene dimer (2-Methyl-1-pentene), cyclopentanone, naphthalene, benzoic acid, caprolactam
MPs61_SV01	SV-2 KAT GIT	Katosi 01 (Dry season)	2-Methyl-2-butene, cyclopentanone, naphthalene,
MPs61_SV02			Cyclopentanone, naphthalene
MPs61_SV03			2,2,3,3,5,6,6-Heptamethylheptane, naphthalene
MPs62_SV03	SV-3 KAT GIT	Katosi 02 (Dry season)	Propylene pentamer (2,4,6,8-Tetramethyl-1-undecene), cyclopentanone, benzoic acid, caprolactam
MPs63_SV02	SV-4 KAT GIT	Katosi 03 (Wet season)	Propylene tetramer (2,4,6-Trimethyl-1-nonene), benzoic acid, caprolactam, 1,20-Heneicosadiene
MPs64_SV01	SV-5 KAT GIT	Katosi 04 (Wet season)	Propylene dimer (2-Methyl-1-pentene), benzoic acid, caprolactam
MPs64_SV03			Propylene tetramer (4,6,8-Trimethyl-1-nonene), benzoic acid, caprolactam
MPs65_SV01 to MPs65_SV04	SV-1 PB GIT	Port Bell 01 (Dry season)	Cyclopentanone, benzoic acid
MPs66_ SV03	SV-2 PB GIT	Port Bell 02 (Dry season)	Propylene dimer (2-Methyl-1-pentene), cyclopentanone, benzoic acid, caprolactam
MPs66_ SV04			Propylene tetramer (2,4,6-Trimethyl-1-nonene), cyclopentanone, benzoic acid, caprolactam
MPs67_ SV02	SV-PB GIT	Port Bell 03 (Wet season)	Propylene dimer (2-Methyl-1-pentene), naphthalene, benzoic acid, caprolactam
MPs67_ SV03			Propylene dimer (4-Methyl-1-pentene), trimer (2,6-Dimethyl-3-heptene), naphthalene, caprolactam, benzoic acid
MPs67_SV04			Propylene trimer (2,3-Dimethyl-2-heptene), caprolactam, cyclopentanone, naphthalene, benzoic acid
MPs68_SV01	SV-PB GIT	Port Bell 04 (Wet season)	Propylene trimer (2,3-Dimethyl-3-heptene), cyclopentanone, naphthalene, caprolactam, benzoic acid

MPs68_SV02			Methyl methacrylate, cyclopentanone, caprolactam, naphthalene, benzoic acid
MPs68_SV03			Propylene trimer (2,4-Dimethyl-1-heptene), tetramers (2,4,6-Trimethyl-1-nonene and 2,2,3,5,6-Pentamethyl-3-heptene), 4-Phenylcyclohexene, caprolactam, benzoic acid
MPs68_SV04			Propylene dimer (2-Methyl-1-pentene), trimers (2,3-Dimethyl-3-heptene and 2,5,5-Trimethyl-2-hexene), tetramers (2,4,6-Trimethyl-1-nonene; 2,4,4,6-Tetramethyl-2-heptene and 2,2,5,5-Tetramethyl-3-hexene), styrene, caprolactam, naphthalene, benzoic acid

Note: Propylene oligomer interferences are indicated in blue. In the analytical disc pair and field codes, SV = *Synodontis victoriae* and PA = *Protopterus aethiopicus*. Samples with polypropylene above the limit of detection (4.24 µg) or limit of quantification (14.13 µg) are shaded.

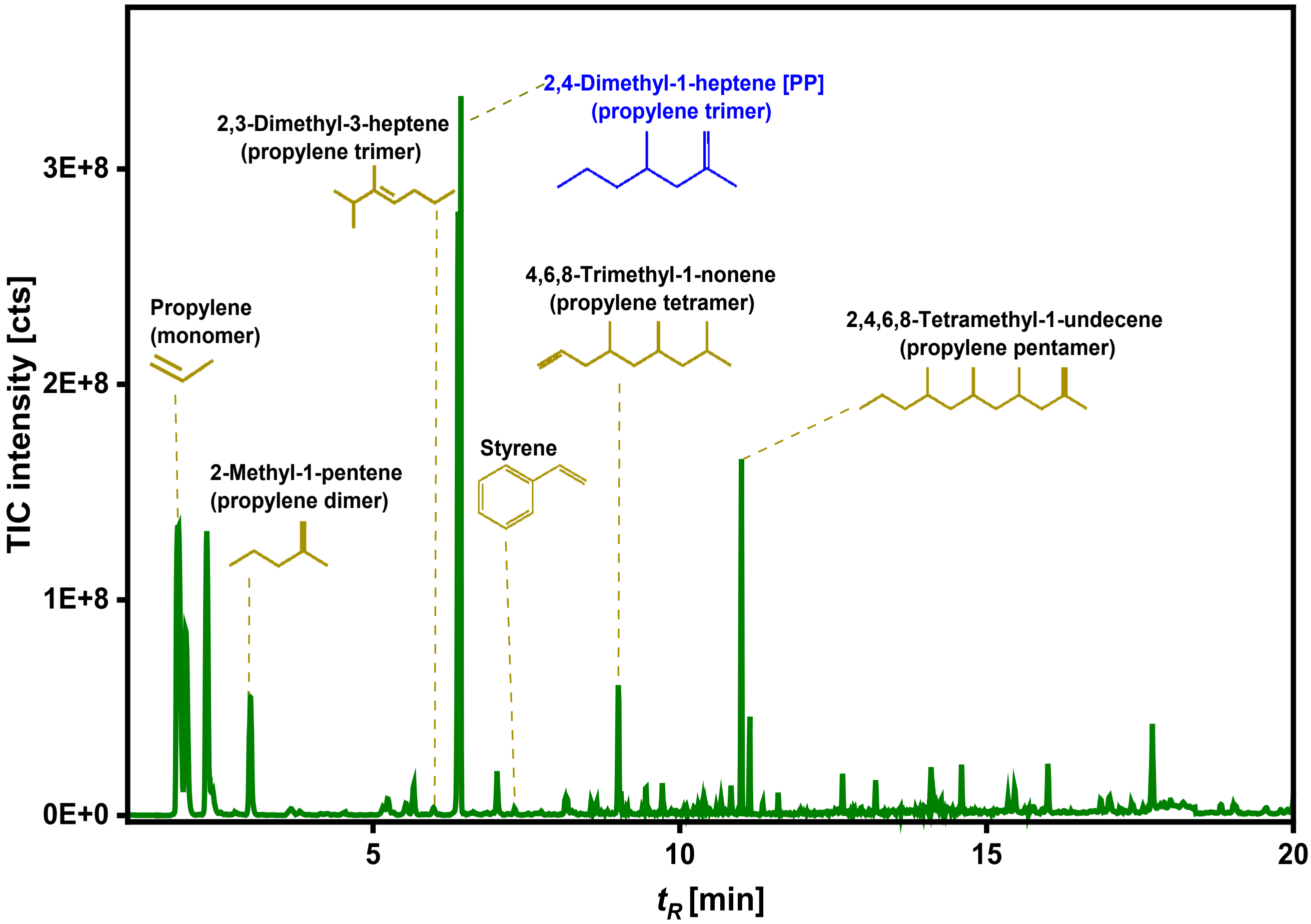

Fig. S7. Pyrogram of a 3D printing filament showing clusters of propylene oligomers detected. Propylene (monomer) was also detected, which was not observed for the fish gastrointestinal tracts.

References

Aisu, R. (2022). Composition and population structure of fish species landed at Katosi and Ggaba landing sites, Lake Victoria Uganda. *BSc Thesis, Makerere University, Kampala*.

Badamasi, I., Odong, R., & Masembe, C. (2020). Gonadal development and intersex condition of marbled lungfish, *Protopterus aethiopicus* (Heckel, 1851) in contaminated sites in Lake Victoria, Uganda. *African Zoology, 55*(4), 292–302. https://doi.org/10.1080/15627020.2020.1811152

Bishai, H. M., & Abu Gideiri, Y. B. (1965). Studies on the biology of genus *Synodontis* at Khartoum. *Hydrobiologia, 26*(1–2), 85–97. https://doi.org/10.1007/BF00142257

Campbell, L.M., Balirwa, J., Dixon, D., & Hecky, R. (2004). Biomagnification of mercury in fish from Thruston Bay, Napoleon Gulf, Lake Victoria (East Africa). *African Journal of Aquatic Science, 29(1)*, 91–96. https://doi.org/10.2989/16085910409503796

Clarke, S. B., Nesbitt, W. A., Efitre, J., Masette, M., & Chapman, L. J. (2022). Elemental composition of small pelagic fishes in three East African lakes: Implications for nutritional security. *Fisheries Research, 256*, 106479. https://doi.org/10.1016/j.fishres.2022.106479

East Africa High Commission. (1959). East African Fisheries Research Organization Annual Report 1958. Retrieved on 8th August 2023 from http://hdl.handle.net/1834/32743

Eccles, D. H. (1992). Field guide to the freshwater fishes of Tanzania, FAO species identification sheets for fishery purposes. In: FAO, Rome, Italy, V, 145 pages.

Froese, R., & Pauly, D. (2016). *Synodontis victoriae*. In: FishBase. June 2016 version. Retrieved on 11th February 2023 from https://www.fishbase.se/summary/9638

Gathu, S. W., Kitaka, N. K., Sitoki, L. M., & Otachi, E. O. (2024). Levels and Classification of Microplastics and Their Impact on the Wellbeing of Selected Commercially Important Fish Species in Kisumu Bay, Lake Victoria. *Bulletin of Environmental Contamination and Toxicology, 113(6)*, 65. https://doi.org/10.1007/s00128-024-03976-w

Goudswaard, K., Witte, F., & Chapman, L. (2002). Decline of the African lungfish (*Protopterus aethiopicus*) in Lake Victoria (East Africa). *African Journal of Ecology, 40*(1), 42–52. https://doi.org/10.1046/j.0141-6707.2001.00335.x

Greenwood, P. H. (1958). Reproduction in the East African Lungfish *Protopterus aethiopicus* Heckel. *Proceedings of the Zoological Society of London, 130*(4), 547–567. https://doi.org/10.1111/j.1096-3642.1958.tb00585.x

Isumbisho, M., Petit, P., Gashagaza, J. B., & Moreau, J. (2011). The feeding habit of the Cyprinidae *Rastrineobola argentea* in its new habitat, lakes Bulera and Ruhondo, two Rwandan lakes (Eastern

Africa). *Knowledge and Management of Aquatic Ecosystems, 403(04)*, 10. https://doi.org/10.1051/kmae/2011044

Manyala, J. O., & Ojuok, J. E. (2007). Survival of the Lake Victoria Rastrineobola argentea in a rapidly changing environment: Biotic and abiotic interactions. *Aquatic Ecosystem Health & Management, 10(4)*, 407–415. https://doi.org/10.1080/14634980701704155

Mlewa, C., & Green, J. (2004). Biology of the marbled lungfish, *Protopterus aethiopicus* Heckel, in Lake Baringo, Kenya. *African Journal of Ecology, 42*(4), 338–345. https://doi.org/10.1111/j.1365-2028.2004.00536.x

Mlewa, C., Green, J., & Simms, A. (2005). Movement and habitat use by the marbled lungfish Protopterus aethiopicus Heckel 1851 in Lake Baringo, Kenya. *Hydrobiologia, 537*, 229–238. https://doi.org/10.1007/s10750-004-3076-3

Mlewa, C. M., Green, J. M., & Dunbrack, R. (2007). Are wild African lungfish obligate air breathers? Some evidence from radio telemetry. *African Zoology, 42(1)*, 131-134. https://doi.org/10.1080/15627020.2007.11407386

Mosille, O. I. I. W., & Mmnoya, J. R. (1988). Reproductive biology of the East African lungfish (P*rotopterus aethiopicus*) in Mwanza Gulf, Lake Victoria. *African Journal of Ecology, 26*(2), 149–162. https://doi.org/10.1111/j.1365-2028.1988.tb00965.x

Muyodi, F. J., Mwanuzi, F. L., & Kapiyo, R. (2011). Environmental Quality and Fish Communities in Selected Catchments of Lake Victoria. *The Open Environmental Engineering Journal, 4*, 54-65. http://dx.doi.org/10.2174/1874829501104010054

Mwanja, M. T., Muwanika, V., Nyakaana, S., Masembe, C., Mbabazi, D., Rutasire, J., & Mwanja, W. W. (2011). Population morphological variation of the Nile perch (*Lates niloticus*, L. 1758), of East African Lakes andtheir associated waters. *African Journal of Environmental Science and Technology, 5(11)*, 941-949. https://doi.org/10.5897/AJEST11.197

Natugonza, V., Ogutu-Ohwayo, R., Musinguzi, L., Kashindye, B., Jónsson, S., & Valtysson, H. T. (2016). Exploring the structural and functional properties of the Lake Victoria food web, and the role of fisheries, using a mass balance model. *Ecological Modelling, 342*, 161–174. https://doi.org/10.1016/j.ecolmodel.2016.10.002

Okedi, J. (1971). Maturity, sex ratio and fecundity of the lung fish (*Protopterus aethiopicus*) from Lake Victoria. *EAFFRO Annual Report*, 17-20.

Olsén, K. H., & Bonow, M. (2023). Crucian carp (*Carassius carassius* (L.)), an anonymous fish with great skills. *Ichthyological Research 70*, 313–331. https://doi.org/10.1007/s10228-022-00892-z

Orlov, A. A., Golubtsov, A. S., Baron, V. D., & Pavlov, D. S. (2015). Bioelectric Fields of the African Marbled Lungfish *Protopterus aethiopicus* (Sarcopterygii: Protopteridae), African (*Heterotis niloticus*) and South American Silver (*Osteoglossum bicirrhosum*) Arowanas (Actinopterygii: Osteoglossidae): Primitive Electrogenesis? *Journal of Ichthyology, 55(6)*, 874–879. https://doi.org/10.1134/S0032945215060120

Poste, A. (2010). Microcystin in Ugandan lakes: Production dynamics, accumulation in fish, and risk evaluation. *PhD Thesis, University of Waterloo, Waterloo, Canada*. Retrieved on 30th March 2024 from http://hdl.handle.net/10012/5691

Seifert, A. W., & Chapman, L. J. (2007). The relationship between temperature and standard rate of metabolism in African lungfish, *Protopterus aethiopicus*, from Uganda. *African Journal of Aquatic Science, 32*, 299–303. https://doi.org/10.2989/AJAS.2007.32.3.10.309

Wandera, S. B. (1990). The exploitation of small pelagic fishes of the great lakes of Africa with reference to the mukene (*Rastrineobola argentea*) fishery of the northern waters of Lake Victoria. In: IAC, Fisheries of the African Great lakes, Papers presented at the International Symposium on resource use and conservation of the African great lakes, Bujumbura, 1989. International Agricultural Centre, Wageningen, Netherlands, pp. 67−74. Retrieved on 30th March 2024 from http://hdl.handle.net/1834/35571

Yongo, E., Manyala, J., Kito, K., Matsushita, Y., Outa, N., & Njiru, J. (2016). Diet of Silver Cyprinid, *Rastrineobola argentea* in Lake Victoria, Kenya. *International Journal of Advanced Research, 4(04)*, 144–149. https://dx.doi.org/10.21474/IJAR01/113

Yongo, E., Olukoye, J., Makame, A., & Chebon, B. (2019). Changes in some biological parameters of the silver cyprinid, *Rastrineobola argentea* (Pellegrin, 1904) in the Nyanza Gulf of Lake Victoria, Kenya. *Lakes and Reservoirs: Research and Management, 24(3)*, 255-258. https://doi.org/10.1111/lre.12281

Yongo, E., & Wairimu, A. (2018). Studies on the Biology of *Synodontis victoriae* in the Nyanza Gulf of Lake Victoria, Kenya. *Fisheries and Aquaculture Journal, 9*(2), 1000244. https://doi.org/10.4172/2150-3508.1000244
